# Graphene in photonic sensing: From fundamentals to cutting-edge applications

**DOI:** 10.1016/j.fmre.2025.08.003

**Published:** 2025-08-21

**Authors:** Muhammad Ali Butt

**Affiliations:** Warsaw University of Technology, Institute of Microelectronics and Optoelectronics, Warsaw 00-662, Poland

**Keywords:** Photonic sensors, Graphene, Surface plasmon resonance, Enhance sensitivity, Sensitivity enhancement, Optical biosensing, Graphene-coated sensors

## Abstract

Graphene-based photonic sensors have drawn considerable attention for their exceptional optical, electrical, and mechanical properties, enabling the development of highly sensitive, fast, and energy-efficient sensor designs. This review examines the significance of graphene-based photonic sensors and their pivotal role in advancing precision sensing across diverse fields. Key types include fiber-optic, surface plasmon resonance (SPR), and waveguide-integrated sensors, each harnessing graphene’s tunable conductivity and broad-spectrum absorption. These sensors offer enhanced sensitivity for detecting gases, biomolecules, and environmental changes, making them invaluable in medical diagnostics, environmental monitoring, and industrial automation. Additionally, graphene's atomic-scale adaptability facilitates miniaturization and integration with flexible substrates, fostering the development of innovative wearable devices. Looking ahead, graphene’s unique electronic and optical properties promise breakthroughs in ultra-sensitive, multifunctional photonic sensors. Graphene-based photonic sensors hold transformative potential across fields requiring rapid, accurate, and compact sensing, paving the way for next-generation technologies in healthcare, smart environments, and beyond.

## 1 Introduction

Photonic sensors are sophisticated devices that harness the interaction between light and matter to detect and measure a wide range of physical, chemical, or biological properties [[1], [2], [3], [4]]. By applying fundamental photonic principles such as absorption, reflection, refraction, and interference, these sensors can capture real-time, high-resolution data with remarkable accuracy and sensitivity [5]. One of the major advantages of photonic sensors is their ability to operate with minimal physical contact with the target medium, making them ideal for applications where traditional contact-based sensors might be less effective or even invasive [[6], [7], [8], [9]]. Photonic sensors are pivotal in numerous sectors, where they meet demands for fast, precise, and reliable measurements [[10], [11], [12], [13]].

One of the most critical applications of photonic sensors is in environmental monitoring [[14], [15], [16]]. These sensors are used extensively to detect pollutants, analyze atmospheric conditions, and monitor changes in water and soil quality [17]. Through photonic sensing, environmental scientists can detect trace gases, particulates, and chemical concentrations at exceedingly low levels, facilitating early detection of environmental changes and compliance with regulatory standards [5,13]. Photonic sensors, for instance, enable real-time monitoring of greenhouse gases, volatile organic compounds, and hazardous particles, which are crucial for assessing air and water quality and managing environmental health [[18], [19], [20]].

In biomedical sensing, photonic sensors are transforming diagnostics and treatment by enabling highly sensitive detection of biological markers and pathogens in bodily fluids [[21], [22], [23]]. For example, optical biosensors can identify minute quantities of biomolecules or pathogens with exceptional specificity, making them invaluable for early diagnosis and disease monitoring. Photonic sensors play a vital role in detecting biomarkers associated with cancer, cardiovascular diseases, and infectious agents, and they facilitate non-invasive or minimally invasive tests, reducing discomfort for patients [24,25]. Wearable photonic sensors are also gaining traction in personalized medicine, where they continuously monitor physiological indicators, such as heart rate, blood oxygen levels, and glucose levels, providing real-time data that can guide patient-specific treatment decisions [10,12,[26], [27], [28]]. Furthermore, WaveFlex biosensor is an innovative optical fiber sensor that leverages localized surface plasmon resonance (LSPR) for the sensitive detection of biomolecules. Its unique design incorporates an S-tapered and waist-expanded structure, enhancing its interaction with target analytes [29]. Functionalization with gold nanoparticles amplifies the plasmonic signal, while cerium oxide nanorods and tungsten disulfide nanosheets increase the sensor's surface area and improve biomolecule binding. This combination results in high sensitivity, low cost, and robust anti-interference capabilities, making the WaveFlex biosensor a promising tool for medical diagnostics and environmental monitoring applications [30].

Another essential application of photonic sensors is in optical communication, where they support the core of fiber-optic networks by detecting and processing data transmitted through light waves [3,30,31]. Due to their exceptional sensitivity to environmental changes, such as temperature, pressure, and mechanical stress, photonic sensors are crucial for ensuring the reliable performance and maintenance of optical communication systems [[32], [33], [34]]. Additionally, photonic sensors are widely applied in industrial process monitoring, where they help optimize safety and efficiency by enabling precise, real-time measurements in challenging environments, such as high-temperature or high-vibration settings that electronic sensors struggle to withstand [14].

Graphene, a single layer of carbon atoms arranged in a two-dimensional honeycomb lattice, has captured significant attention for its unparalleled properties and transformative potential across numerous fields [35,36]. Among graphene's most remarkable characteristics is its exceptional electrical conductivity, derived from its unique electronic structure, where mobile charge carriers move with minimal resistive loss due to its zero bandgap and ultrahigh carrier mobility. This makes graphene one of the best conductors and positions it as a top candidate for various electronic and optoelectronic applications, including high-speed transistors, flexible electronics, and photodetectors [37,38]. Transition metal dichalcogenides (TMDs) [39], such as MoS₂, WS₂, and MoSe₂, are 2D materials that exhibit a direct bandgap in their monolayer form, which makes them particularly promising for optoelectronic applications like photodetectors, light-emitting devices, and photonic sensors. The direct bandgap of TMDs allows for efficient light absorption and emission, which is a critical feature for enhancing the sensitivity and responsiveness of photonic sensors [40,41]. In contrast, graphene, being a zero-bandgap material, does not naturally exhibit light-emitting or light-absorbing properties to the same extent as TMDs. However, graphene's high carrier mobility and tunable optical conductivity make it an ideal candidate for photonic sensing when coupled with other materials or engineered to form hybrid systems. The key advantage of graphene in such systems lies in its ability to serve as an ultra-fast, low-resistance conductive layer that can be integrated with other optoelectronic components for enhanced sensing performance [42,43].

Perovskites, particularly organic-inorganic hybrid perovskites (such as CH₃NH₃PbI₃), have also attracted significant interest for their excellent light absorption, ease of fabrication, and exceptional photodetector properties [44]. Perovskites exhibit a tunable bandgap and high charge-carrier mobility, which makes them highly suitable for use in photonic sensing devices, including light sensors and solar cells [45]. While perovskites offer remarkable advantages in terms of light absorption efficiency, their stability, especially under environmental stress (e.g., moisture and UV degradation), remains a significant challenge. Graphene, by comparison, exhibits exceptional mechanical stability and resistance to environmental degradation, making it an ideal material for encapsulating and protecting other materials, including perovskites, in hybrid sensor devices [46].

Graphene also possesses an exceptionally large specific surface area, with one gram able to cover approximately 2,630 square meters, making it highly effective for applications requiring high sensitivity, such as chemical and biological sensing. This high surface area enhances its reactivity and ability to adsorb a wide range of molecules, making graphene a powerful tool for capturing trace quantities of analytes [47,48]. Additionally, graphene exhibits extraordinary mechanical strength, with a Young’s modulus of around 1 TPa, making it one of the strongest materials available [46]. This strength allows graphene to be used in flexible, durable devices that can withstand significant mechanical stresses, an asset for sensors operating in demanding conditions. One of graphene’s most beneficial features for photonic applications is its high optical transparency [49]. With approximately 97.7% light transmittance in the visible spectrum, graphene allows most light to pass through with minimal obstruction, making it ideal for use in photonic sensors where undisturbed light interaction with the analyte is essential. This transparency, combined with its chemical stability and biocompatibility, positions graphene as a uniquely versatile material in the field of photonics, with promising applications in both environmental and biomedical sensing, as well as optical communication [47,50,51].

The incorporation of graphene as a coating material for photonic sensors is motivated by its potential to significantly improve sensor performance and expand application possibilities [52]. One of the primary benefits of graphene coating is its ability to enhance the sensitivity of photonic sensors [53]. The high surface area and chemical reactivity of graphene facilitate the efficient adsorption of target molecules, leading to pronounced changes in the optical properties of the sensor, such as shifts in refractive index or absorption spectra. This enhanced sensitivity is particularly advantageous for detecting low-concentration analytes, as required in trace gas sensing and biosensing of biomarkers at low concentrations, enabling early detection and real-time monitoring with heightened accuracy.

Graphene's unique properties also enable the miniaturization of photonic sensors. Due to its atomic-scale thickness and robust mechanical strength, graphene allows for the creation of compact, lightweight, and flexible sensors without compromising durability or performance [54]. Miniaturized sensors are critical for portable and wearable applications, such as environmental monitors and health diagnostics, where space and power efficiency are essential. Graphene-coated photonic sensors are ideal for developing such compact devices, which are not only easy to deploy but also more cost-effective, expanding the scope and accessibility of advanced sensing technologies. Furthermore, graphene imparts multi-functionality to photonic sensors, broadening their applicability across diverse fields. For example, graphene's high conductivity enables electro-optic modulation, allowing for the development of tunable photonic sensors that can respond to both optical and electrical stimuli [55,56]. This dual capability is invaluable for creating adaptive, smart sensors that can operate in dynamic or multi-signal environments, such as in industrial automation or smart wearable devices [57]. Graphene's biocompatibility also allows for safe use in biomedical sensors, opening possibilities for advanced diagnostic tools and therapeutic monitoring systems that interact safely with biological tissues and fluids [[58], [59], [60]].

## 2 Fundamental properties of graphene and its interaction with photonic structures

**Fig. 1:**
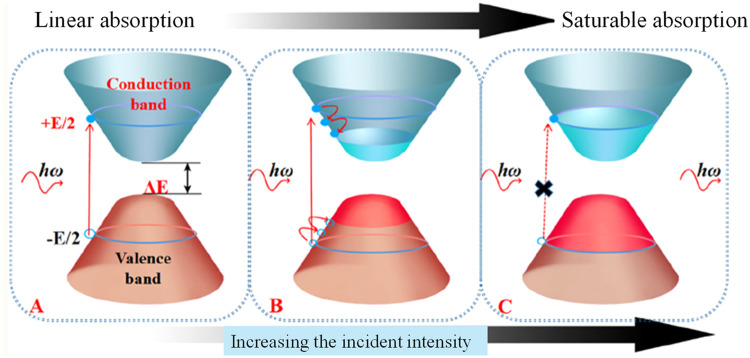
**Saturable absorption is explained through the Pauli blocking principle [**62**]**.

Saturable absorption in photonic devices based on two-dimensional (2D) materials is primarily governed by two mechanisms: sub-bandgap absorption and the Pauli blocking effect [61]. The choice of mechanism depends on the energy of the incident light relative to the material’s bandgap, denoted as ΔE (illustrated in Fig. 1) [62]. When the incident photon energy is lower than ΔE, direct absorption does not occur. Instead, absorption may be facilitated by alternative processes such as defect states, sub-bandgap transitions, or nonlinear effects like two-photon absorption. These indirect pathways allow the material to interact with light below its bandgap energy. In contrast, when the incident photon energy exceeds ΔE, the saturable absorption mechanism is primarily attributed to Pauli blocking. At low optical intensities, standard linear absorption takes place: electrons in the valence band absorb incoming photons and are excited into the conduction band, forming electron-hole pairs. These carriers rapidly thermalize and follow a Fermi-Dirac distribution. Subsequently, they relax to equilibrium through intraband phonon scattering. As the intensity of incident light increases, more energy states near the band edges become occupied due to the higher density of photocarriers. Once these states are filled, further photon absorption is inhibited, as dictated by the Pauli exclusion principle. This saturation of available states leads to a decrease in absorption, allowing photons to pass through the material hallmark of saturable absorption. Understanding and controlling the bandgap ΔE is thus critical in engineering ultrafast photonic devices using 2D materials, as it directly influences the absorption behavior and overall device performance [62].

Graphene’s extraordinary electrical and optical properties have positioned it as a groundbreaking material in photonics and sensor technology. Structurally, graphene is a single layer of carbon atoms arranged in a hexagonal lattice, granting it an ultra-thin yet robust structure with remarkable electronic characteristics, such as tunable optical conductivity [63], extremely high carrier mobility [64], and an extensive absorption spectrum [43]. One of the most significant properties of graphene is its band structure near the Dirac points, where the valence and conduction bands converge [65]. This unique configuration allows graphene to host charge carriers that behave like massless Dirac fermions, enabling exceptionally high carrier mobilities. At room temperature, graphene's carrier mobility reaches approximately 10,000 cm²/V·s and can exceed 200,000 cm²/V·s at low temperatures, making it ideal for applications requiring rapid charge transport and high sensitivity, such as photonic sensors [66].

Graphene’s optical conductivity can be dynamically tuned, a feature rarely found in conventional materials [67]. By altering its Fermi level—either through electrostatic gating or chemical doping—the conductivity of graphene can be controlled across a wide frequency range, from the visible to the THz spectrum. This tunability enables graphene to interact flexibly with incident light, an asset in photonic sensing that allows engineers to tailor sensor responses to specific wavelengths. Graphene's light absorption, despite its single-atom thickness, is notably around 2.3% of incident light in the visible range, which is remarkable for such an ultrathin material. For applications requiring stronger light interaction, graphene layers can be stacked, or hybrid materials can be used to amplify absorption, enhancing graphene’s effectiveness in diverse photonic devices. Combined with its high electron mobility, this optical tunability provides unprecedented control over light-matter interactions, a critical factor for designing advanced photonic sensors with fast response times and high sensitivity [68].

Li et al. proposed a novel dual-channel localized surface plasmon resonance (LSPR) sensor that integrated silver nanodisks (Ag-disks), a zinc sulfide (ZnS) film, and a two-dimensional graphene layer acting as a key interfacial material [69]. This hybrid structure enabled the excitation of LSPR modes between the Ag-disks and the ZnS surface, facilitating two distinct sensing channels within the visible light spectrum. The sensor's performance was experimentally assessed from two perspectives: bulk refractive index sensitivity and surface sensitivity. Incorporating graphene led to a notable enhancement in performance, achieving a bulk sensitivity of 271 nm/RIU—a 56% increase compared to the version without graphene. Furthermore, the sensor demonstrated improved sensitivity to minor changes in its immediate surface environment. This performance boost was attributed to the near-field enhancement induced by the graphene layer, which amplified LSPR excitation efficiency. Overall, the proposed sensor design offered a promising framework for dual-channel plasmonic sensing and laid a strong foundation for future development of LSPR-based devices incorporating 2D materials [69].

The interaction between graphene and surface plasmon resonance (SPR) is transformative for enhancing the sensitivity and precision of photonic sensors [[70], [71], [72]]. SPR is a phenomenon where incident light induces collective oscillations of electrons, or plasmons, on the surface of metals like gold or silver, resulting in a confined electromagnetic field near the surface [73]. This localized field is exceptionally sensitive to refractive index changes near the metal, which is crucial for detecting minute changes in the environment [74,75]. When graphene is integrated with SPR sensors, it significantly amplifies the SPR effect due to its high optical absorption and tunable conductivity, making it an ideal partner for SPR-based applications [[76], [77], [78], [79]]. Kravets et al. fabricated and studied hybrid metal-dielectric-graphene heterostructures using oxides (Al_2_O_3_, HfO_2_, ZrO_2_) and Si_3_N_4_, combined with plasmonic metals Cu and Ag, for SPR biosensing [80]. Incorporating dielectric/graphene layers significantly improved SPR characteristics, including reflectivity, phase changes, resonance width, and sensitivity to refractive index variations. The maximum spectral sensitivity exceeded 30,000 nm/RIU for Cu/Al_2_O_3_, Ag/Si_3_N_4_ bilayers, and Cu/dielectric/graphene three-layer structures in the near-infrared range. These sensitivities were 5–8 times higher than those of bare Cu or Ag films. Additionally, the resonance width was reduced, and an unexpected blueshift in the resonance spectral position was observed. This blueshift, along with the enhanced SPR sensitivity, was attributed to stationary surface dipoles that generated a strong electric field at the thin dielectric/graphene layer [80].

The interaction between graphene and the plasmonic field enhances the electromagnetic field’s intensity, allowing for higher sensor sensitivity. Graphene’s ability to support charge carriers at high densities, coupled with its high surface area, makes it particularly effective at confining electromagnetic fields near the sensor surface [81]. Moreover, because graphene’s Fermi level can be adjusted via external gating, the SPR signal can be dynamically modulated to adapt the sensor’s response to different analytes or environmental changes. This capability allows for real-time adjustment of SPR conditions, an asset in creating sensors with adaptable sensitivity profiles and selective detection capabilities. Additionally, graphene’s integration helps to reduce plasmonic mode damping, resulting in sharper and more pronounced SPR peaks, which are vital for achieving high-resolution detection. Graphene’s biocompatibility and large surface area also facilitate functionalization with biomolecules, enabling selective and high-sensitivity SPR-based sensors for biosensing, chemical detection, and environmental monitoring [82]. By combining the distinct properties of graphene with SPR, researchers have developed highly sensitive platforms capable of detecting trace amounts of biological or chemical substances, making graphene-enhanced SPR a valuable technology for medical diagnostics and environmental applications. Table 1 summarizes the characteristics of graphene-coated devices and their potential applications.

**Table 1:** **Characteristics of graphene-coated sensors and applications**.

Aspect	Description	Applications
Sensitivity	High sensitivity due to graphene’s excellent optical and electrical properties.	Biosensing, chemical detection, environmental monitoring [[83], [84], [85], [86]]
Optical Properties	Strong optical absorption, broad spectral response, and tunable bandgap.	Optical communication, IR and UV photodetectors [[87], [88], [89]]
Electrical Conductivity	Excellent conductivity enhances signal-to-noise ratio.	High-speed data transmission, photodetection [90]
Surface Area	High surface-to-volume ratio increases interaction with analytes.	Gas sensing, chemical sensing, biomolecule detection [91,92]
Flexibility	Flexible and mechanically robust, allowing use on curved surfaces.	Wearable sensors, flexible electronics [57,93,94]
Transparency	Optical transparency makes it suitable for integration with optical systems.	Transparent displays, photovoltaic cells [95,96]
Biocompatibility	Biocompatible and suitable for in vivo applications.	Medical diagnostics, implantable biosensors [[97], [98], [99]]
Thermal Stability	High thermal stability allows use in harsh environments.	Aerospace, industrial monitoring [100,101]
Chemical Stability	Resistant to many chemicals, increasing sensor longevity.	Environmental monitoring, chemical processing [50,100,102]
Photodetection Efficiency	High efficiency for detecting photons over a broad spectrum.	Imaging, communication, solar cells [[103], [104], [105], [106]]
Light Modulation	Graphene can modulate light effectively in optical waveguides.	Optical switches, modulators in photonic circuits [[107], [108], [109]]

Defects in graphene can be introduced through various processes, such as chemical doping, mechanical strain, or high-energy irradiation, leading to vacancies, grain boundaries, or functional groups like hydroxyl or carboxyl groups [110]. These defects can significantly alter the electronic properties of graphene, affecting charge transport and carrier mobility, which in turn can influence sensor sensitivity and response time. For instance, defect sites can act as adsorption centers for target molecules, enhancing sensitivity but possibly introducing non-specific binding or reducing the selectivity of the sensor. The type, density, and distribution of defects play a pivotal role in determining how graphene interacts with its environment, especially when used in sensors for gas detection, biosensing, or chemical analysis. Furthermore, the functionalization of graphene to tailor its properties for specific sensor applications is another area in need of deeper investigation. Functionalization strategies aim to modify the graphene surface to enhance its reactivity or provide specific recognition sites for target molecules [111]. Common functionalization methods include covalent bonding with organic molecules, non-covalent interactions with biomolecules or polymers, and the use of metal nanoparticles to improve the performance of graphene-based sensors [112]. These functional groups can also help mitigate the impact of defects by passivating unwanted reactive sites or promoting selective binding with specific analytes. However, the optimization of these functionalization strategies, such as controlling the degree of functionalization and ensuring uniform coverage across the graphene surface, remains a significant challenge. Understanding how different functional groups interact with graphene’s defects and how they influence sensor performance is key to the development of more efficient and reliable sensors in various fields, including environmental monitoring, medical diagnostics, and industrial applications [113].

## 3 Types of graphene-coated photonic sensors and their applications

This section provides a detailed analysis of various types of photonic sensors enhanced with graphene coatings, highlighting their unique properties, mechanisms, and potential applications. Table 2 presents a comprehensive overview of the recently proposed and notable advancements in graphene-based photonic sensors.

**Table 2:** **Recently proposed graphene-based photonic sensors for different applications**.

Sensor structure	Applications	Type of study	Sensitivity	Reference
Hybrid plasmonic-photonic sensor	Biochemical	Numerical	6682 nm/RIU	[175]
Graphene-based composite structure	Pressure	Experimental	17.86 nm/kPa	[176]
Graphene-coated photonic surface-wave resonance	Refractive index	Numerical	7023 nm/RIU	[177]
1-D topological photonic system	Refractive index			[178]
Photonic crystal cavity	Humidity	Experimental	3.9 dB/%RH	[179]
Copper-graphene-based Photonic crystal fiber	Refractive index	Numerical	2000 nm/RIU	[180]
Graphene-based optical sensor	Flow sensing of a single-cell	Numerical	4.3 × 10^−7^ mV/RIU	[181]
Graphene-based photonic crystal	Refractive index	Numerical	1178.6 nm/RIU	[182]
Graphene-based MS absorber	Refractive index and temperature	Numerical	981 nm/RIU; −0.23 nm/°C	[48]
Graphene oxide-based optical fiber sensor	Humidity	Experimental	0.1036 dB/%RH	[183]

### 3.1 Surface plasmon resonance (SPR) sensors

SPR is a sophisticated optical sensing technique that leverages the excitation of surface plasmons which is a collective oscillation of conduction electrons at the interface between a metal and a dielectric medium [114]. This phenomenon occurs when polarized light interacts with the free electrons on a thin metal film, usually gold, deposited onto a glass prism. When incident light is directed at a specific angle, known as the resonance angle, it induces surface plasmons, creating an evanescent electromagnetic wave confined to the metal-dielectric interface. This evanescent wave is highly sensitive to changes in the local refractive index within the immediate vicinity of the metal surface [115].

The SPR sensor is typically configured in a Kretschmann geometry, where polarized light enters a high-refractive-index prism and undergoes total internal reflection at the prism-metal interface [116]. The resonance condition is achieved when the wavevector of the incident photons matches that of the surface plasmons, leading to maximum coupling between the light and the surface plasmon field. This results in a distinct drop in reflected light intensity, detectable by a photodetector. Any perturbation, such as the binding of biomolecules to the sensor surface, modifies the local refractive index and shifts the resonance angle or wavelength. This shift is proportional to the analyte concentration, enabling quantitative and real-time monitoring of molecular interactions without requiring fluorescent or radioactive labels. SPR's high sensitivity to surface-bound molecules renders it a powerful tool for biochemical assays, including antibody-antigen binding, DNA hybridization, and protein-protein interactions.

Jamil et al. investigated a graphene-based SPR biosensor that enhanced urea detection efficiency through high adsorption capabilities [117]. Utilizing the Kretschmann configuration, renowned for its effectiveness in plasmon excitation, the research explored the influence of adding a thin molybdenum disulfide (MoS₂) layer on top of graphene, deposited on a gold (Au) substrate in the SPR setup. Simulations are conducted using the FDTD method to assess biosensor performance, particularly focusing on sensitivity and the FWHM of the SPR spectrum. Experiments were carried out at wavelengths of 670 nm and 785 nm for targeted urea detection. By adjusting the refractive index of the sensing layer from 1.335 to 1.347, the study assessed response variations across different molarities. Results indicated maximum sensitivities of 230°/RIU at 670 nm and 173.16°/RIU at 785 nm, highlighting the biosensor’s high potential for precise urea detection. This graphene-MoS₂-Au structure demonstrated significant promise for sensitive biochemical applications, offering improved performance for SPR biosensing technology.

The integration of graphene, a monolayer of sp²-bonded carbon atoms arranged in a honeycomb lattice, onto the metal layer of an SPR sensor substantially amplifies sensitivity due to graphene’s unique electronic and structural properties [118]. Graphene’s high surface-to-volume ratio, exceptional electrical conductivity, and capability to form π-π interactions enhance the interaction between the evanescent field and analyte molecules. This heightened interaction occurs because graphene enhances the local field intensity near the metal surface, effectively amplifying the sensor's response to refractive index changes. Furthermore, graphene's intrinsic biocompatibility and chemical versatility facilitate the adsorption and retention of biomolecules, resulting in stronger and more precise analyte binding. Thus, the signal intensity is considerably enhanced, enabling the detection of ultralow concentrations of analytes, down to the femtomolar or even attomolar range in optimized conditions, far surpassing the capabilities of conventional SPR sensors [82,119].

Graphene-coated SPR sensors also exhibit an extended detection range due to the unique electronic properties of graphene, such as high charge carrier mobility and robust light-matter interactions. The graphene layer acts as an additional dielectric medium that modulates the penetration depth of the evanescent field, allowing it to reach analytes situated farther from the metal interface. This extended field range makes it feasible to detect larger biomolecular complexes, such as protein aggregates, vesicles, or even cells, without compromising sensitivity. Additionally, the presence of graphene enhances the robustness of the plasmonic resonance across a broader refractive index spectrum, making it adaptable to diverse sensing environments. This extension of detection depth and versatility significantly expands the utility of SPR sensors, enabling applications in fields requiring precise detection over larger spatial domains, including cell-based assays and environmental toxin detection.

**Fig. 2:**
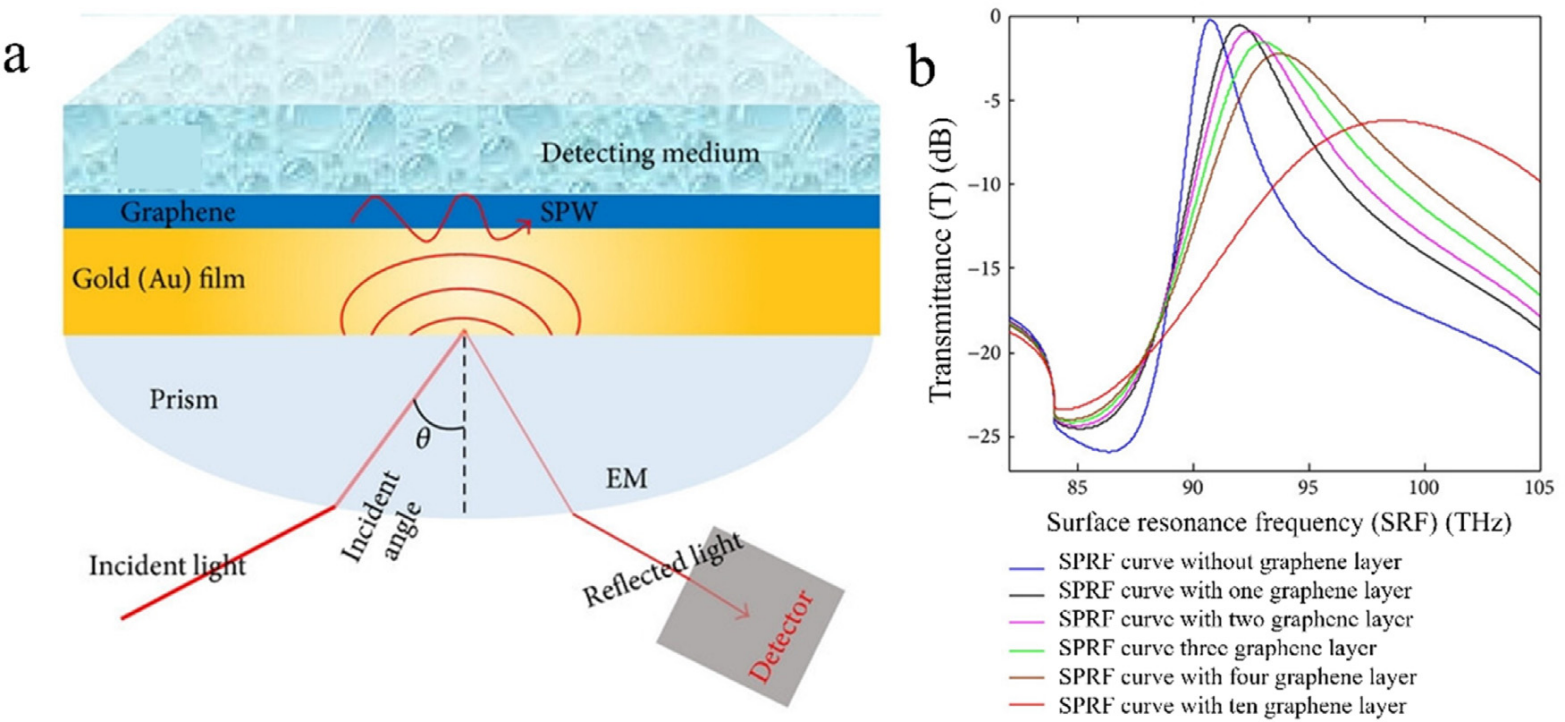
**(a) Diagram of the 4-layer SPR biosensor model: prism, a 50 nm gold layer, a graphene layer (0.34L nm, where L represents the number of graphene sublayers), and the sensing medium (water) [**120**], (b) T-SRF curves for both the standard biosensor (*L* = 0) and the graphene-coated biosensor (*L* = 1, 2, …, 10) before DNA molecule adsorption [**120**]**.

Hossain et al. introduced a numerical model of an SPR biosensor designed to detect DNA hybridization by analyzing resonance frequency characteristics (RFC) [120]. The sensor incorporated graphene as a biomolecular recognition element (BRE) alongside the Au layer (Fig. 2a), which generated a precise SPR response. A numerical analysis compared the transmission-resonance frequency (T-SRF) characteristics curve of the sensor both with and without a graphene sublayer, before DNA molecule addition, commonly referred to as the bare sensor. This comparison is illustrated in Fig. 2b. The SPR frequency for the sensor without graphene is 91.70 THz while introducing a single layer of graphene shifted the SPR frequency to 92.65 THz. This frequency increase (ΔSRF) of 0.95 THz toward the right side of the T-SRF curve was attributed to the addition of each graphene layer, which significantly enhanced sensor sensitivity [120].

Simulations indicated that RFC variations are minimal for mismatched DNA strands but show notable shifts for fully complementary strands. Graphene enhanced the immobilization between target and probe DNA, using its unique optochemical properties to improve hybridization detection. The sensor effectively distinguished DNA hybridization from single-nucleotide polymorphisms (SNPs) by detecting differences in RFC and peak transmittance, providing accurate identification of genetic variations. Additionally, the inclusion of graphene sublayers significantly boosted sensitivity, achieving a 95% improvement over conventional SPR sensors. Sensitivity analysis highlighted the benefits of the graphene sublayer, positioning this frequency-based SPR sensor as a promising tool for precise monitoring of biomolecular interactions [120].

Graphene’s remarkable surface chemistry and functionalization potential further enhance the selectivity of SPR sensors. Graphene’s basal plane can be easily functionalized with a variety of biomolecules, including peptides, antibodies, aptamers, and small organic molecules, via covalent or non-covalent modifications. For instance, by introducing functional groups like carboxyl (-COOH), amine (-NH₂), or hydroxyl (-OH) groups, graphene can be tailored to exhibit high affinity for specific classes of analytes. This functionalization capability is highly advantageous in achieving selective recognition of target analytes, especially in complex biological matrices where specificity is critical [70]. For example, graphene-functionalized SPR sensors can be designed for the selective detection of nucleic acids, proteins, or small biomolecules, with a high signal-to-noise ratio and reduced cross-reactivity [121].

For instance, Irfan et al. presented a dual-function plasmonic perfect absorber sensor with high efficiency, designed using lithium niobate (LiNbO₃) and graphene layers for applications in refractive index and thermal sensing [48]. The sensor’s structure was intentionally simplified to enable easy fabrication, consisting of a LiNbO₃ substrate, a quartz layer, a thin graphene film, four gold nanorods, and a centrally positioned nanocavity within each unit cell. The placement of the nanocavity allowed enhanced penetration of electromagnetic energy into subsurface layers, thereby maximizing the sensor’s response. The sensor achieves a strong reflection response of 99.9%, ensuring high detection capability without requiring specialized operational conditions. Numerical simulations demonstrated that the sensor operates effectively within a biomedical refractive index range of 1.33 to 1.40, achieving a sensitivity of 981 nm/RIU and a figure of merit (FOM) of 61.31 RIU⁻¹, highlighting its suitability for precise biochemical sensing. Furthermore, the addition of a polydimethylsiloxane (PDMS) layer allowed the sensor to function as a thermal sensor, with a thermal sensitivity of −0.23 nm/ °C. These findings underscore the sensor’s versatility and high sensitivity, making it an excellent candidate for both refractive index and temperature-based biomedical applications [48].

### 3.2 Fiber optic sensors

Fiber optic sensors operate on the principle of light transmission through optical fibers to detect variations in environmental or physical parameters, such as temperature, strain, pressure, or chemical composition [3]. These sensors exploit the unique properties of optical fibers, which transmit light via total internal reflection within a high-refractive-index core surrounded by a cladding layer with a lower refractive index [122]. This core-cladding configuration allows light to propagate over long distances with minimal attenuation, making fiber optic sensors highly suitable for remote sensing applications in which signal fidelity is essential. The sensing mechanism in fiber optic sensors is based on modulating specific light parameters such as wavelength, intensity, phase, or polarization in response to external perturbations [123]. For example, in fiber Bragg grating (FBG) sensors, a periodic refractive index structure is inscribed within the fiber core. This grating reflects a specific wavelength, known as the Bragg wavelength, which shifts in response to strain or temperature changes. By monitoring this wavelength shift, the sensor can quantitatively determine variations in the surrounding environment. Similarly, interferometric fiber optic sensors rely on the phase difference between light waves travelling through multiple optical paths, allowing the detection of minute changes in environmental conditions with high precision.

Fiber optic sensors offer several key advantages, including immunity to electromagnetic interference, high sensitivity, and the ability to function under extreme environmental conditions [124]. Furthermore, they support multiplexing, enabling simultaneous measurement at multiple points along a single optical fiber. These properties make fiber optic sensors particularly valuable for applications in structural health monitoring, medical diagnostics, aerospace, and oil and gas industries. However, the detection capabilities of fiber optic sensors can be significantly enhanced by optimizing light-matter interactions within the fiber, particularly through advanced material coatings such as graphene, which can improve both sensitivity and signal-to-noise ratio (SNR) [125].

Coating fiber optic sensors with graphene improves critical performance metrics by augmenting light-matter interaction and enhancing SNR. This improvement is primarily due to graphene's high optical absorption, electrical conductivity, and unique interaction with electromagnetic waves across a broad range of frequencies [126]. Graphene coatings enhance the light-matter interaction in fiber optic sensors by increasing the evanescent field sensitivity, which is essential for detecting subtle changes in the surrounding environment [127]. When light propagates through a graphene-coated fiber, the evanescent field extends slightly beyond the fiber core and interacts with the graphene layer. Due to its high surface area and strong photon absorption capabilities, graphene significantly increases the fiber's sensitivity to external perturbations [128]. This heightened sensitivity allows for more precise detection of environmental changes, including temperature, mechanical strain, and the presence of specific chemical species, enabling the fiber optic sensor to achieve higher detection accuracy and resolution [129].

Furthermore, the integration of graphene coatings enhances the sensor's SNR, a crucial parameter in sensor performance that defines the ratio of the desired signal relative to background noise. Graphene’s high electrical and thermal conductivity contributes to noise reduction by dissipating thermally induced fluctuations that often degrade signal quality. This property is especially beneficial in high-temperature environments, where conventional fiber optic sensors may suffer from significant noise-related interference. Additionally, graphene's atomic-scale interaction with light reduces signal attenuation, maintaining signal clarity and intensity over extended transmission distances. This effect is particularly valuable in applications requiring long-range measurements, where signal integrity is paramount [130]. Huang et al. introduced a platform that incorporates a hollow-core fiber with a graphene coating [127]. Experimental findings revealed that anti-resonant reflecting guidance was significantly enhanced, producing distinct and periodic lossy dips within the transmission spectrum. This setup achieves a sensitivity of −365.9 dB/RIU and a detection limit as low as 2.73 × 10⁻⁶ RIU by monitoring the intensity of these lossy dips. This design was promising for highly sensitive applications in chemistry, medical, and biological research.

Fluorescence resonance energy transfer (FRET), with its inherent selectivity, is widely used in chemical and biomedical analysis. However, traditional FRET systems face limitations in achieving both high sensitivity and compact design. Yao et al. addressed this by presenting a novel "FRET on Fiber" approach, where a partially reduced graphene oxide (prGO) film was integrated into a fiber-optic modal interferometer [131]. This prGO layer served as both a FRET fluorescence quencher and a sensitive cladding layer, enabling precise optical phase measurements in response to refractive index changes. In this setup, target analytes trigger fluorescence recovery with high selectivity alongside measurable optical phase shifts with outstanding sensitivity. The prGO-coated fiber-optic interferometer demonstrated excellent detection capabilities, with limits as low as 1.2 nM for metal ions, 1.3 μM for dopamine, and 1pM for single-stranded DNA (ssDNA). This "FRET on Fiber" configuration merged FRET functionality with fiber-optic sensing in a compact, highly sensitive, and selective platform, making it ideal for real-time monitoring in environmental, chemical, and biomedical applications [131].

**Fig. 3:**
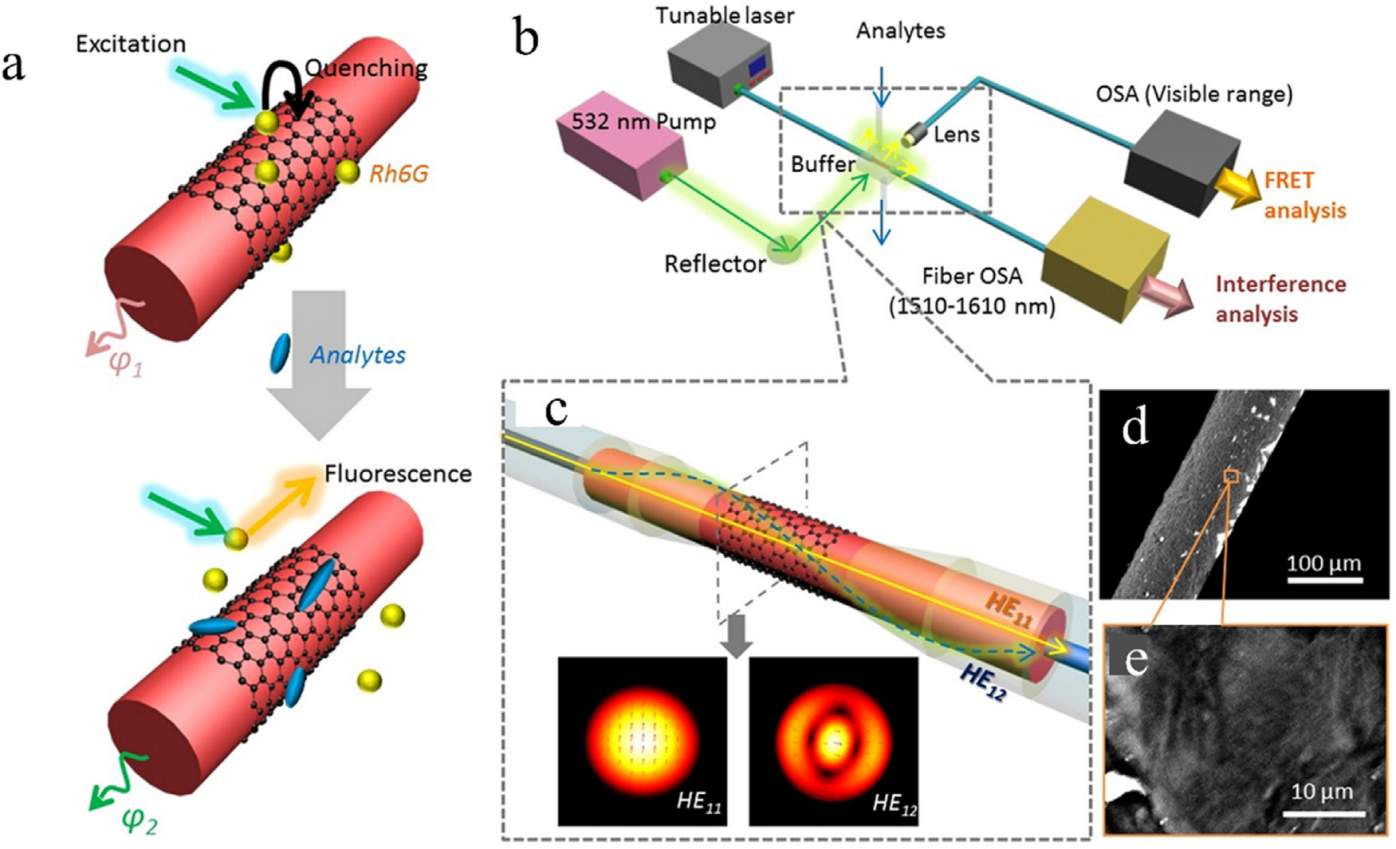
**(a) the principle of the graphene-based “FRET on Fiber” system is illustrated, where both fluorescence recovery and optical phase modulation occur on the prGO film [**131**], (b), the detection setup includes two distinct channels: one dedicated to fluorescence detection and another for all-fiber interference phase measurement. The prGO-coated fiber-optic interferometer is immersed in a 0.1 mL buffer, allowing analytes to be introduced and removed [**131**], (c) the probe structure is detailed, showing the prGO film (black hexagons) around the etched multimode fiber (MMF) section. The interferometer facilitates interference between HE_11_ and HE_12_ modes, with the HE_12_ mode amplified in the prGO-coated region, as modeled by the FEM method in COMSOL [**131**], (d) SEM image displays the prGO film as a dark layer, with (e) offering an enlarged view of this layer from (d) [**131**]**.

The prGO-based "FRET on Fiber" detection system is illustrated in Fig. 3a. For biochemical sensing, an experimental setup was developed (Fig. 3b) with a prGO-coated fiber-optic modal interferometer as the core sensor. This interferometer was constructed by placing a section of multimode fiber between two single-mode fibers, creating a singlemode-multimode-singlemode (SMS) configuration, which was then coated with a prGO film, as shown in Fig. 3c. Figs. 3d,e present SEM images of the prGO-coated fiber. In comparison to monolayer graphene produced via CVD, the prGO, reduced from GO, has a rougher surface and appears darker [131].

**Fig. 4:**
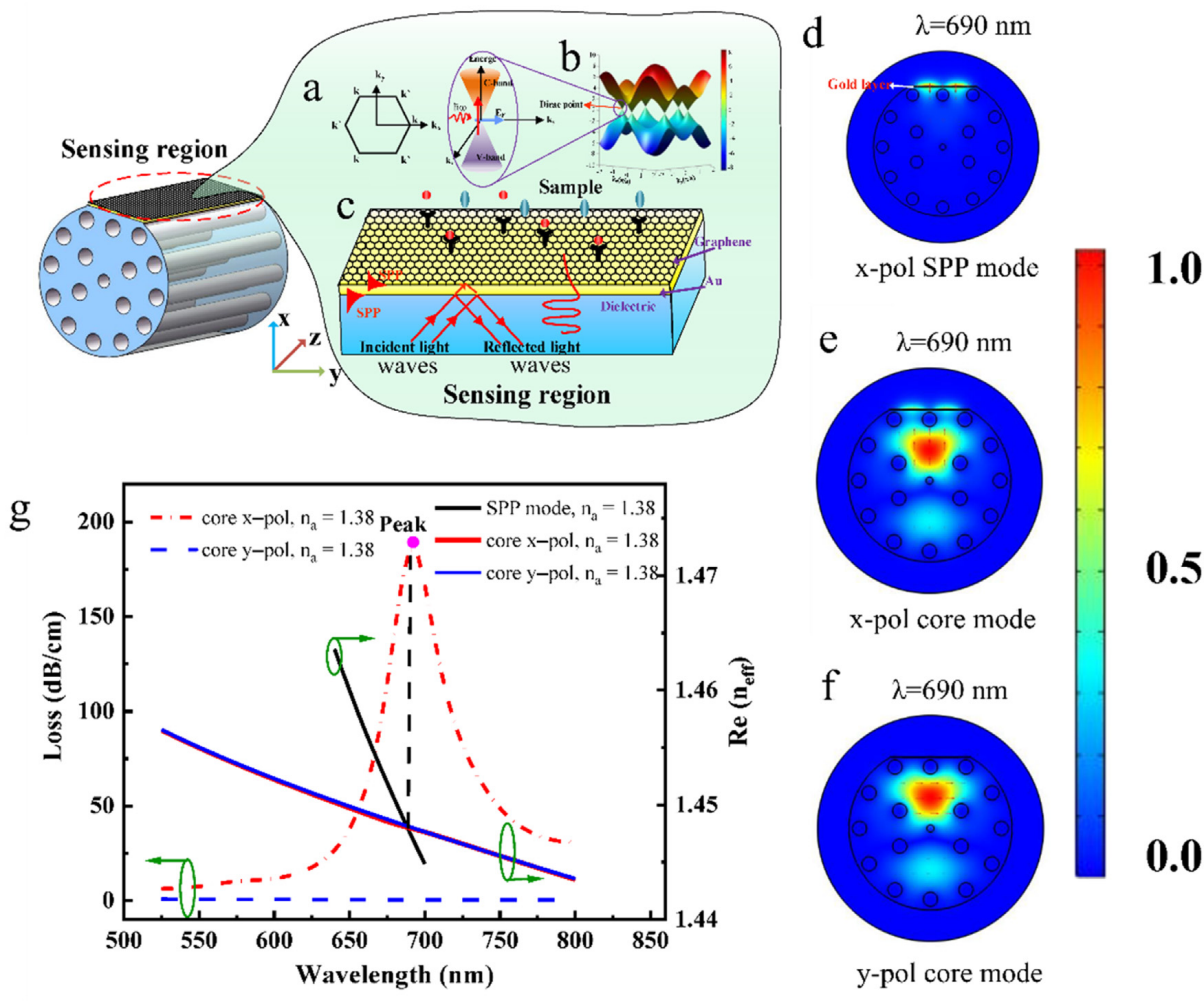
**The SPR mechanism for a d-shaped PCF sensor is depicted as follows: (a) The Brillouin zone of the graphene lattice structure [**137**], (b) The linear dispersion curve representing single-layer graphene’s band structure, and (c) A schematic of the graphene-gold layer's sensing process [**137**]. Mode field distribution at 685 nm is shown for (d) x-polarized SPP mode, (e) x-polarized core mode, and (f) y-polarized core mode, (g) The dispersion relationship between the fundamental core-guided mode and the SPP mode is illustrated [**137**]**.

In contrast to conventional optical fibers, photonic crystal fibers (PCFs) feature an array of air holes in the cladding, running continuously along the fiber axis [132,133]. This unique structure grants PCFs remarkable versatility and a range of advantageous characteristics, such as the ability to support single-mode transmission over a broad wavelength range, high nonlinearity, significant birefringence, expansive mode field area, easy material infiltration, low transmission loss, and tunable dispersion [134]. These qualities make PCFs ideal for applications requiring compactness, easier integration, and cost efficiency, and they have demonstrated potential as alternatives to traditional prisms in optical systems [135,136]. Yang et al. introduced a graphene-gold coated photonic crystal fiber (PCF) sensor optimized for operation in the visible light spectrum (Fig. 4c) [137]. By implementing a side-polished d-shaped surface over the defect region of the PCF’s periodic air hole structure, the design significantly enhanced the evanescent field interaction. The graphene layer atop the gold coating not only stabilized the adsorption of biomolecules but also enhanced the propagation constant of the surface plasmon polariton (SPP), contributing to improved sensor sensitivity. The sensor achieved exceptional sensitivity, with a maximum wavelength sensitivity of 4200 nm/RIU, an amplitude sensitivity of 450 RIU⁻¹, and a refractive index resolution of 2.3 × 10⁻⁵ RIU across the refractive index range of 1.32–1.41. This research highlights a promising approach for developing next-generation biosensors with significantly enhanced sensitivity and stability.

To enhance the sensitivity of the graphene-Au SPR sensor integrated into a d-shaped PCF, a thorough understanding of graphene’s optical characteristics is crucial. Graphene is a single atomic layer of carbon atoms, structured in a two-dimensional honeycomb lattice through sp² hybridization, as shown in Fig. 4(a). The components of the wave vector *k_x_* and *k_y_ * are symmetric in this lattice, resulting in a linear energy dispersion at specific points within the Brillouin zone, where the conduction and valence bands meet. This linear dispersion forms a "Dirac cone," characterizing graphene’s unique electronic properties in which electrons behave like massless Dirac fermions, exhibiting relativistic behavior.

Fig. 4b presents the band structure of graphene, with a close-up of the Dirac point. This structure highlights graphene’s semimetallic nature with a zero-band gap, where the conical conduction and valence bands intersect, enabling enhanced electron mobility and sensitivity critical for SPR applications. Fig. 4d-f illustrate the distribution of the mode field, providing a visual representation of coupling strength. Fig. 4g presents the dispersion curves for the SPP and core modes [137].

### 3.3 Waveguide-Based sensors

Photonic waveguide sensors leverage the propagation of light through a confined waveguide structure to detect subtle changes in the surrounding environment, with applications spanning chemical, biological, and environmental sensing [138,139]. In these sensors, light is transmitted through a high-index material (core), such as silicon, silicon nitride, or polymer, which is surrounded by a cladding material with a lower refractive index. This difference in refractive index ensures that light is confined within the core via total internal reflection, although a portion of the light, known as the evanescent field, extends slightly into the surrounding environment [140,141]. This evanescent field provides a mechanism for interaction with external analytes, as changes in the refractive index near the waveguide surface alter the properties of the transmitted light, allowing for precise detection [142].

Common designs include Mach-Zehnder interferometers (MZI) [19,143,144] and ring resonators [[145], [146], [147]], each optimized for distinct sensing capabilities and levels of integration into photonic circuits. Due to the high sensitivity of the evanescent field to external changes, photonic waveguide sensors enable real-time, label-free detection of analytes, allowing for their use in miniaturized and integrated sensing platforms. However, traditional waveguide sensors may experience limitations in sensitivity and signal amplification, particularly when detecting analytes at low concentrations or in complex matrices. To address these limitations, graphene has been introduced as a material to augment waveguide sensing capabilities, offering enhancements in both sensitivity and signal strength. One key mechanism by which graphene enhances waveguide sensors is through signal amplification resulting from its strong optical absorption and high interaction with electromagnetic fields [148]. When incorporated into a waveguide structure, graphene increases the interaction between the evanescent field and analytes in contact with the graphene layer. Graphene’s high optical conductivity enables it to support surface plasmon polaritons (SPPs) at visible and near-infrared wavelengths, which intensify the evanescent field interaction, leading to stronger analyte-induced signal variations. This enhanced interaction results in a more pronounced shift in the light's properties upon analyte binding, enabling the detection of lower analyte concentrations compared to traditional waveguides [139].

Additionally, graphene enhances detection sensitivity through its inherent conductivity and high chemical reactivity [149]. The incorporation of graphene into the waveguide introduces additional charge carriers that respond to local changes in the environment, especially upon analyte binding. This reactivity makes graphene an excellent material for electro-optical sensing applications, wherein changes in the analyte's electrical properties translate to measurable optical shifts in the guided light. Binding events between analytes and the graphene layer cause local changes in graphene’s conductivity and refractive index, which subsequently influence the optical properties of the waveguide mode. This process allows for highly sensitive detection capabilities, often at levels that exceed the sensitivity of conventional waveguide sensors without graphene integration [150].

Furthermore, graphene’s optical tunability via electrostatic gating allows for additional control over the sensor’s response, making it possible to adjust the sensor’s sensitivity and selectivity for specific analytes or environmental conditions [151]. This feature is particularly beneficial for applications requiring precise control over sensing parameters, such as in biosensing or chemical detection, where analyte concentration and binding affinity are critical [152]. Moreover, graphene’s compatibility with a variety of substrates and waveguide materials enables its integration into diverse sensor architectures, including interferometric sensors, resonators, and SPR platforms [153]. Karthikeyan et al. presented a method for detecting moisture in transformer oil using an optical waveguide made with GO [149]. A GO film was applied onto an SU-8 polymer channel waveguide through drop-casting. For polarized light at a 1550 nm wavelength, the sensor achieved strong absorption of TE light. The device’s TE-polarized light absorption was measured across varying water levels in the oil, from 16 to 21 ppm, including free water. Results showed that TE transmission power increased linearly with rising water content, with a power change of +0.90 dB per ppm. Finite element method (FEM) simulations supported the findings, confirming that this sensing mechanism provides quick response and high sensitivity.

**Fig. 5:**
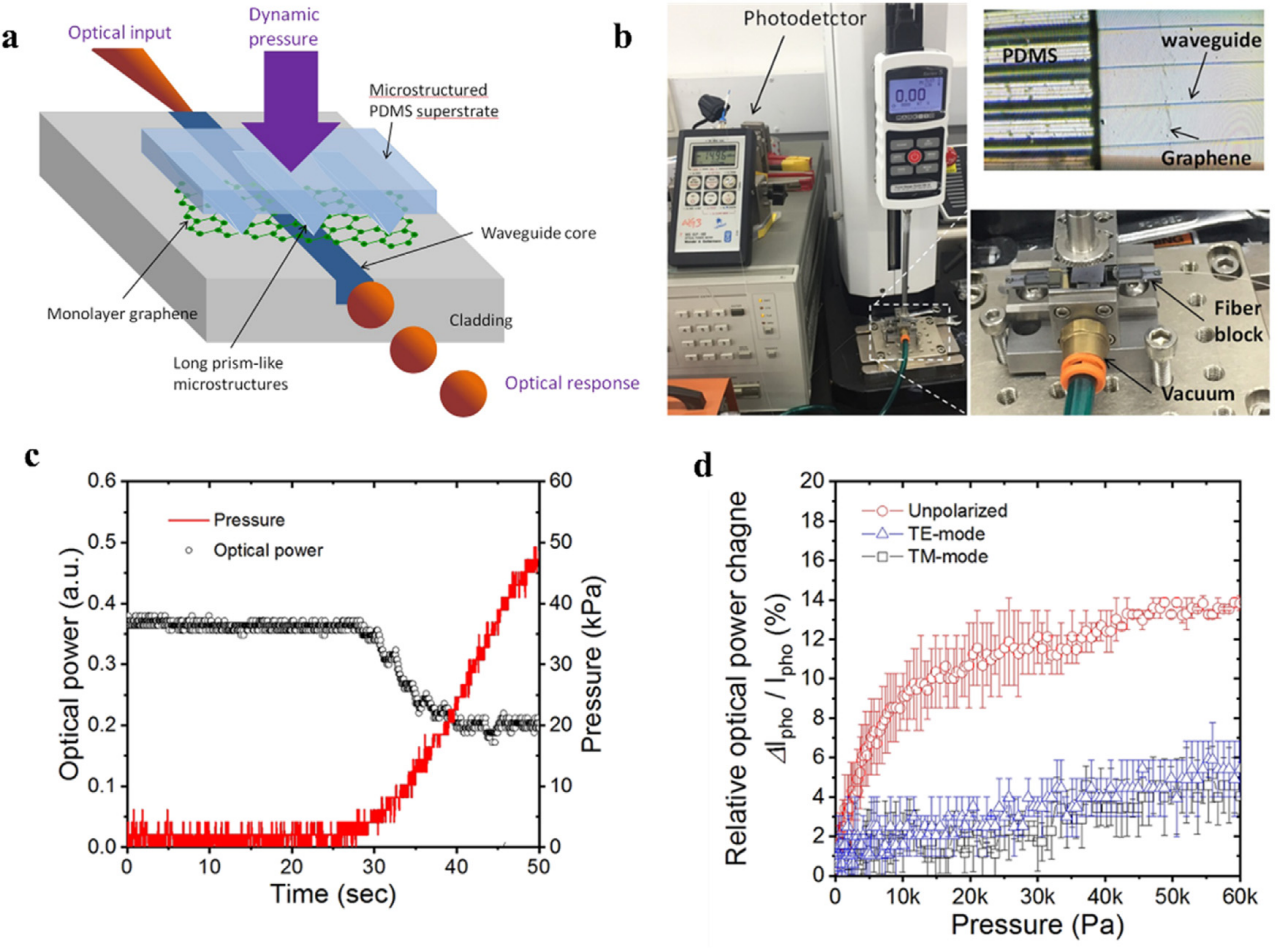
**(a) Top-down view of a graphene-based optical waveguide sensor [**153**], (b) Measurement setup for the graphene-based optical waveguide tactile sensor, which is securely placed on a vacuumed sample stage. A photodetector measures the optical output power, and a microscope image shows a precise alignment of the prism-like structure with the waveguide core [**153**], (c) Output light intensity and applied pressure over time. As pressure increases, the optical output power decreases, reaching saturation at 40 kPa [**153**], (d) Change in relative optical power with pressure. Higher pressure reduces output power, with a larger change in the TE mode than the TM mode due to the graphene’s polarization-sensitive absorption [**153**]**.

Directional coupler-based optical tactile sensors offer various benefits, but their wider application is hindered by a critical requirement: the refractive index of the upper superstrate must be equal to or higher than that of the optical waveguide core. To bypass this constraint, a graphene-enhanced optical waveguide tactile sensor was proposed and experimentally validated by Kim et al. as shown in Fig. 5a [153]. In this design, the lateral deformation of a low-index, prism-like microstructure on an elastomer superstrate played a central role in optically sensing mechanical pressure. By controlling the deformation area, the contact between the waveguide core, graphene, and polydimethylsiloxane (PDMS) can be fine-tuned, thus adjusting the light absorption by graphene. This adjustment remained effective even when the superstrate’s refractive index was lower than the waveguide core. The sensor demonstrated a precise, real-time response to repeated pressing and releasing, tracking multiple steps of mechanical pressure with the aid of a piezoelectric motor. This graphene-based tactile sensor overcomes the limitations of traditional directional coupler designs, enabling broader material compatibility and expanded application potential [153].

Fig. 5(b) illustrates the setup with a Mark-10 force gauge (ESM 303 tester and M5–10 gauge). The tactile sensor was secured on a vacuumed stage, and its optical output was measured by a photodetector. Vertical force was applied by moving a bar on the gauge, pressing on the PDMS layer with an attached flat plate for even pressure, aided by a silicon piece on the PDMS. The input and output fibers were firmly attached to maintain alignment during force application. A microscope image (top right inset) shows the graphene layer on the waveguide, with the prism structure precisely positioned on the waveguide core [153]. Fig. 5(c) shows the output light intensity and the corresponding vertical pressure over time. When no pressure was applied, output power remained nearly constant, with minor fluctuations due to slight instability in the laser diode, which was controlled by a laser diode and thermoelectric cooler (Newport). As pressure was gradually applied, the output power began to decrease, eventually stabilizing at 30 kPa, with no further decrease observed beyond 40 kPa. Fig. 5d displays the relative change in optical power, indicating the difference between output power with and without applied pressure. As pressure increased, output power decreased, leading to an increase in relative optical power change. This occurred because higher pressure broadens the deformed PDMS microstructure, intensifying the field at the waveguide core-graphene-PDMS interface. At pressures above 40 kPa, no further increase in optical interaction occurred due to the limited field diameter of the guided light [153].

### 3.4 Wearable sensors

Wearable sensors have become increasingly integral to modern healthcare, fitness tracking, and even environmental monitoring [154,155]. These devices continuously collect data from the body or surrounding environment, offering real-time insights that can significantly improve health management, athletic performance, and safety. They are typically embedded into everyday items such as wristbands, clothing, or patches that can be worn on the body [10,27,156,157]. Sensors integrated into these devices track metrics like heart rate, temperature, movement, respiration, and sweat composition. Such wearables can offer users continuous health monitoring, allowing for immediate detection of abnormal changes and trends over time, potentially preventing critical health issues [158].

The performance of wearable sensors is contingent on their ability to be both accurate and durable. However, traditional materials used for sensor construction, like metals or polymers [159,160] face limitations in terms of flexibility, sensitivity, and longevity, especially when subjected to the mechanical stresses and environmental conditions associated with being worn continuously on the body. To address these challenges, innovative materials like graphene have been increasingly employed to enhance the capabilities of wearable sensors. When graphene is used to coat wearable sensors, it unlocks a host of performance improvements that traditional materials simply cannot match. These enhancements lead to more accurate data collection, longer wearability, and more efficient sensor operation.

Graphene-coated wearable sensors significantly improve the sensitivity of the devices [57]. This is particularly crucial for applications such as medical diagnostics, where detecting small fluctuations in data is vital. Sensors that monitor metrics like blood glucose levels, heart rate irregularities, or brain activity can achieve far greater precision when graphene is used as a coating. The material’s exceptional electrical conductivity allows for more accurate signal detection, enabling sensors to detect even the slightest variations in physiological parameters, thus offering a higher degree of reliability and accuracy [93].

One of the primary challenges of wearable sensors is ensuring they are durable yet comfortable to wear. Traditional sensors made from rigid materials may become uncomfortable or degrade over time due to repeated bending, stretching, or exposure to moisture [161]. Graphene-coated sensors excel in this regard due to their ability to maintain mechanical integrity while being flexible enough to conform to the body. This means that graphene-coated sensors can endure long periods of continuous wear without losing their functionality, making them ideal for applications where long-term monitoring is needed, such as chronic disease management or fitness tracking [162].

Another significant benefit of graphene-coated sensors is their reduced power consumption. Wearable devices that monitor health parameters typically rely on batteries, and battery life is a major concern for users who need the device to operate over long periods without frequent recharging [163]. The high conductivity of graphene reduces the energy required for data transmission and sensor operation, allowing for more energy-efficient devices. In some cases, this can lead to wearables that can operate for longer periods on a single charge, or even utilize energy harvesting techniques (like body heat or motion) to stay powered [164,165].

A major advantage of graphene is its versatility in integrating multiple functions into a single sensor [166]. Wearables that require the monitoring of a broad range of physiological indicators such as heart rate, skin temperature, movement, and sweat composition benefit from graphene's ability to support multiple sensor types on a single substrate. By coating or integrating graphene into the sensor array, multiple types of data can be captured and transmitted efficiently [167]. This multi-sensing capability is crucial for applications like personalized healthcare or athletic performance analysis, where a wide variety of parameters need to be monitored in real time [168].

For wearable sensors that remain in continuous contact with the skin such as ECG patches or sweat sensors, biocompatibility is a vital concern [169]. Graphene-coated sensors do not irritate the skin or cause allergic reactions, which can be a problem with some metals or polymers. This makes them especially useful for medical-grade wearables that require constant skin contact. The material is also highly breathable, which is important for comfort during prolonged use. This characteristic is crucial for ensuring user compliance in wearable healthcare applications, where sensors must be worn for extended periods to gather accurate and consistent data [52].

In recent years, there has been a growing focus on laser-induced graphene (LIG), a high-performance material with immense potential across various fields, including energy storage, ultra-hydrophobic water applications, and electronic devices [170,171]. LIG has also shown great promise in the area of human motion and posture tracking through the use of flexible sensing materials. Xing et al. investigated the evolution of surface morphology and performance of LIG created with varying laser energy accumulation times [172]. The performance of LIG-based flexible wearable sensors was further assessed for accurate detection of human motion and posture. Experimental results revealed that the LIG sensors demonstrated excellent flexibility and mechanical performance, particularly when the laser energy accumulation was optimized over three cycles. These sensors exhibited remarkable features, including high sensitivity (∼41.4 Gauge Factor), low detection limits (0.05%), fast response times (∼150 ms for activation, ∼100 ms for recovery), and exceptional stability even after 2000 s under strains of 1.0% and 8.0%. These findings underscore the significant potential of LIG-based flexible sensors for monitoring human motion, wrist pulse rates, and eye blinking patterns.

**Fig. 6:**
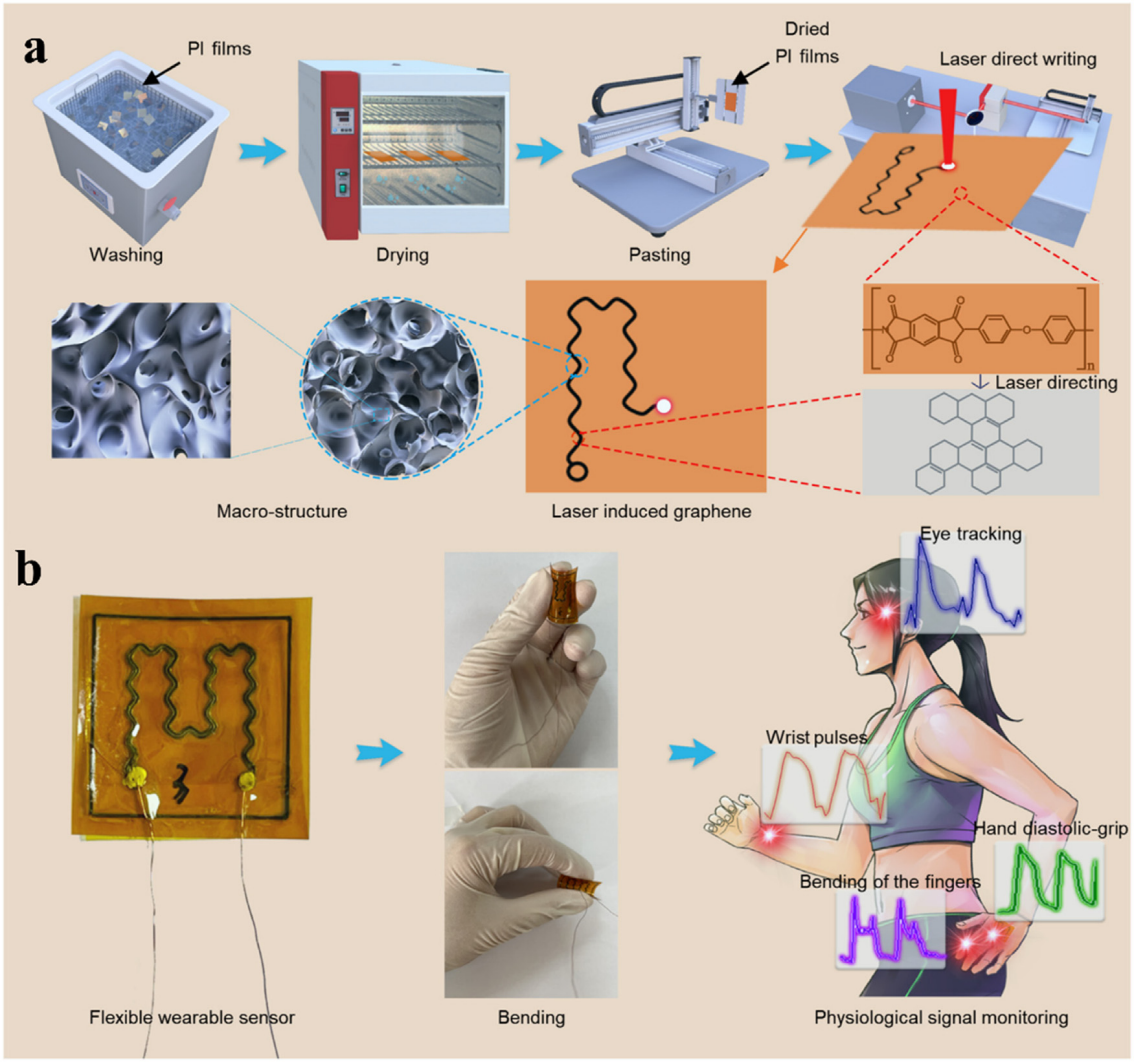
**Overview of LIG devices and their applications: (a) Process of fabricating LIG films [**172**], (b) Construction and application of LIG [**172**]**.

The preparation process for laser-induced graphene (LIG) is illustrated in Fig. 6. Initially, polyimide (PI) films (2.5 mm × 2.5 mm) were thoroughly cleaned with deionized water in an ultrasonic cleaner, and then dried in a blow-drying oven at 50 °C for 2 h (Fig. 6a). After drying, the PI films were attached to a 3D moving stage with PET tape and scanned with a CO₂ infrared laser using these settings: 12.4 W power, 20 kHz pulse repetition frequency, and a scanning speed of 105 mm/s. This method produced a serpentine-shaped LIG pattern over a 1.5 mm × 1.5 mm area. With repeated scans, the LIG surface developed a porous microstructure. Fig. 6b shows that this LIG material can be used to fabricate flexible wearable sensors encapsulated with a 50 μm layer of PI tape. These sensors are highly flexible, adaptable to various shapes, and can be directly attached to the skin with PET tape. They were designed to monitor body posture, pulse rate, and finger movements, enabling real-time tracking of physiological data.

**Fig. 7:**
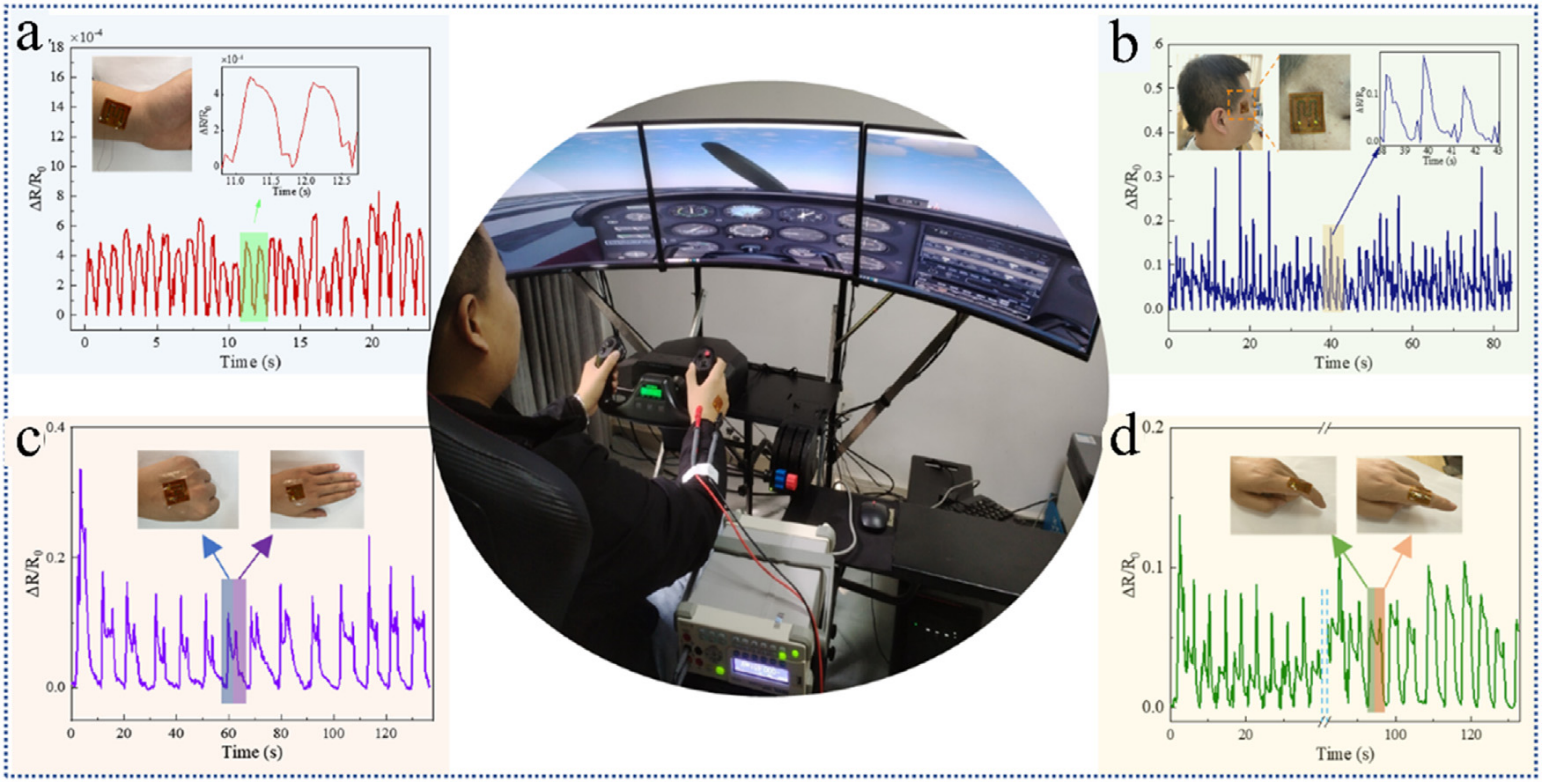
**LIG-based flexible strain sensor for body detection and motion capture in pilots: (a) Pulse detection, (b) Blink detection, (c) Hand grip and stretch test, (d) Finger curvature detection [**172**]**.

Flexible strain sensors, as an emerging technology, have shown great promise for the highly sensitive detection of subtle physiological signals [173,174]. When attached to the human wrist, these sensors can capture pulse vibration frequencies in real-time. This capability is crucial for early disease detection, health monitoring, and analyzing heart rate variability. Flexible strain sensors provide a convenient, accurate, and comfortable alternative to traditional methods like electrocardiography (ECG) and blood pressure monitoring, making them especially valuable for wearable health trackers and individuals with cardiovascular conditions. Xing and colleagues demonstrated that placing a flexible strain sensor on the wrist allowed it to conform closely to the skin, reducing external interference and minimizing measurement errors. Fig. 7a presents the results of wrist pulse detection, highlighting the sensor's effectiveness in capturing small physiological signals. Overall, these sensors provide an efficient, precise, and user-friendly approach to monitoring human physiology.

A flexible strain sensor placed at the corner of the eye successfully detected subtle eye movements without direct contact with the ocular surface, avoiding pressure or interference. This non-invasive design is well-suited for monitoring eye health and studying eye movement control. As illustrated in Fig. 7b, the sensor registered resistance changes corresponding to different blink forces, providing valuable data. When applied to the back of the hand or fingers, the sensor also monitors finger flexibility and gestures, offering potential applications in rehabilitation, sports performance tracking, and gesture recognition technology. Analyzing finger strain patterns, enhanced the accuracy of tracking finger motions and postures, improving rehabilitation therapies and human-computer interaction. Figs. 7c,d highlight the sensor's stability during hand relaxation and finger bending [172].

## 4 Fabrication techniques for graphene-coated photonic sensors

Successfully integrating graphene into photonic sensor systems is critical to preserving its intrinsic properties and ensuring optimal sensor performance. Several techniques have been developed for graphene integration, each tailored to balance quality, scalability, and device compatibility:

### 4.1 Chemical vapor deposition (CVD)

CVD is a primary method for producing high-quality, large-area graphene films. In this process, graphene is synthesized on metal substrates (commonly copper) through the thermal decomposition of carbon-based gases, such as methane, at high temperatures [184]. The resulting graphene layer is then transferred to the target photonic sensor substrate, typically using a polymer support layer like poly(methyl methacrylate) (PMMA) that is later removed. CVD-grown graphene films are particularly advantageous for photonic sensors because of their superior electrical and optical quality, large-scale uniformity, and low defect density. However, CVD involves some challenges, including potential contamination and defects introduced during the transfer process, which can impact sensor performance. Advances in CVD techniques are continually being developed to minimize these issues and to enable large-scale, cost-effective production of high-quality graphene [185,186].

**Fig. 8:**
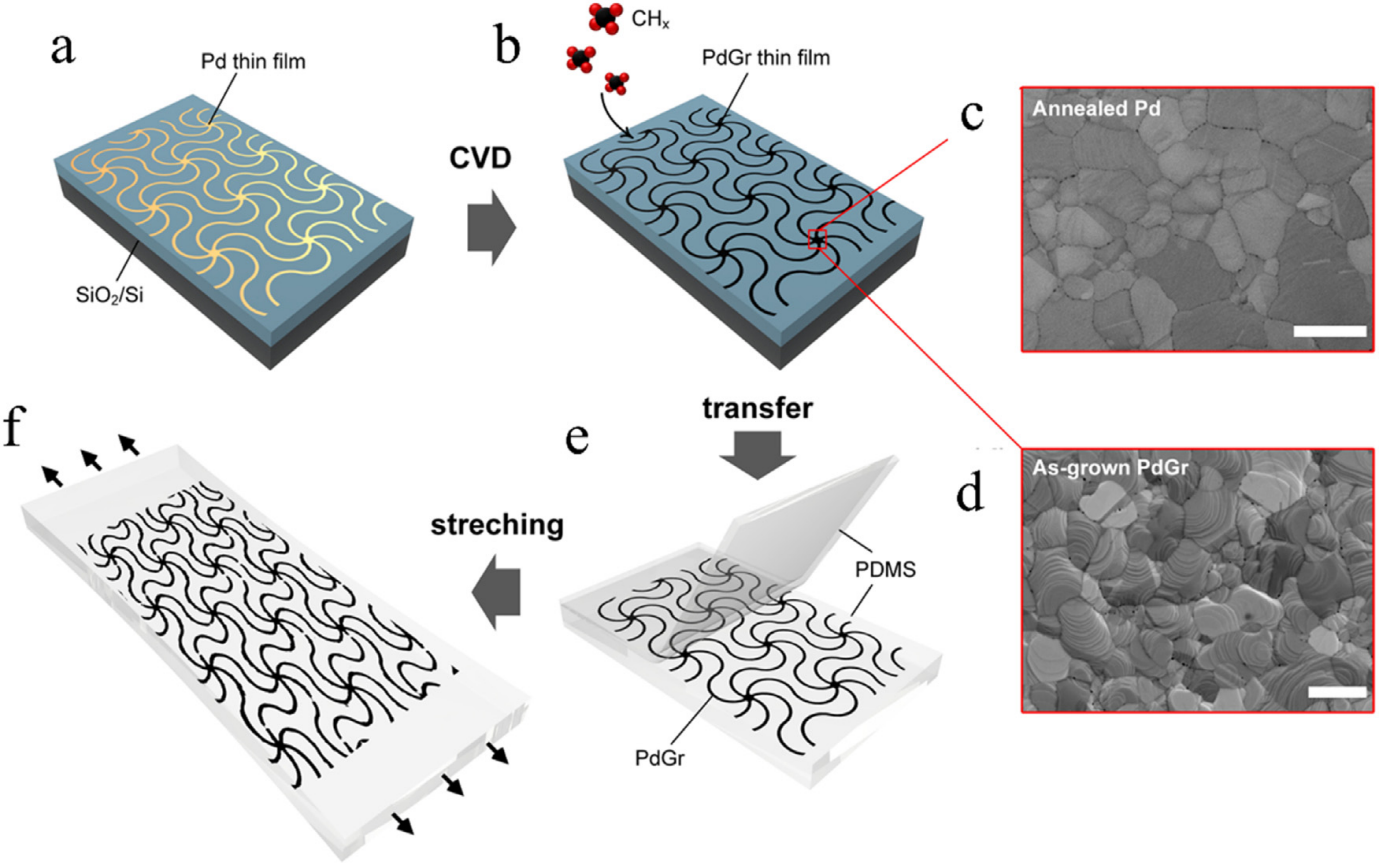
**(a) Illustration of a wavy thin-film network (TFN) in a ‘horseshoe’ design on a SiO₂/Si substrate, (b) Diagram of CVD-grown graphene-metal TFN on the SiO₂/Si substrate, (c, d) SEM images showing the surface morphology of annealed Pd and graphene-coated Pd (PdGr) thin films, before and after graphene growth, respectively, (e) Illustration of the CVD-grown PdGr TFN after transfer and embedding into a flexible PDMS layer, (f) Diagram of the TFN with a PDMS backing under uniaxial stretching [**187**]**.

Conductive metal films patterned on flexible elastomeric substrates form mesh structures ideal for flexible electronic interconnections across diverse applications. Although conventional bottom-up methods like sputtering and material growth effectively produce thin films for rigid electronics, preserving electrical conductivity in sub-micron metal films under extreme deformation or repeated mechanical stress remains challenging. Zhang and colleagues introduced a method to improve the electromechanical durability of nanometer-scale palladium films by applying a conformal graphene coating through in-situ CVD [187]. The continuous graphene layer markedly improved the film’s damage tolerance, resistance to electro-mechanical fatigue, and fracture durability, leveraging the inherent stiffness of graphene and the robust graphene-metal interface formed by CVD. To fabricate CVD-grown PdGr nanocomposite interconnects, a palladium network was patterned on a SiO₂ (300 nm)/Si substrate using photolithography and deposited via sputtering. This Pd network acted as the catalyst for subsequent graphene growth, as depicted in Fig. 8 a, b. The as-deposited palladium thin-film network (TFN) underwent annealing at 600  °C in a helium atmosphere for one hour to relieve residual stress from sputtering, which also reduced grain boundary density (Fig. 8c). The surface morphology of the as-grown PdGr, shown in Fig. 8d, displays reconstructed features like curved steps and terraces within each grain, indicating strong Pd-Gr adhesion. After graphene synthesis, the PdGr TFN can be transferred and encapsulated within a thin layer of polydimethylsiloxane (PDMS), as illustrated in Fig. 8 e,f [187]. PdGr films exhibited stable electrical resistance even under strains exceeding 60% and show extended fatigue life within strain ranges up to 20%. The beneficial effects of graphene were particularly pronounced in films under 300 nm, especially at high strains. These findings aligned with the thin-film electro-fragmentation model and the Coffin-Manson relationship, underscoring the potential of CVD-grown graphene composites in enhancing the durability and electromechanical resilience of flexible electronics [187].

### 4.2 Transfer methods

Various transfer techniques, both wet and dry, are also employed to integrate graphene into photonic devices [188]. Wet transfer is a common approach in which the growth substrate is etched away, allowing a polymer scaffold to support the graphene layer as it is moved to the new substrate [189]. Dry transfer techniques, such as direct lamination or stamp transfer, eliminate the need for liquid etchants, which can introduce impurities or structural imperfections in the graphene layer [190]. Dry transfer is especially beneficial for applications where purity and structural integrity are paramount, such as in ultra-sensitive photonic sensors. These methods also enable the placement of graphene onto flexible or non-planar substrates, broadening the scope of applications in wearable or flexible photonic sensors.

**Fig. 9:**
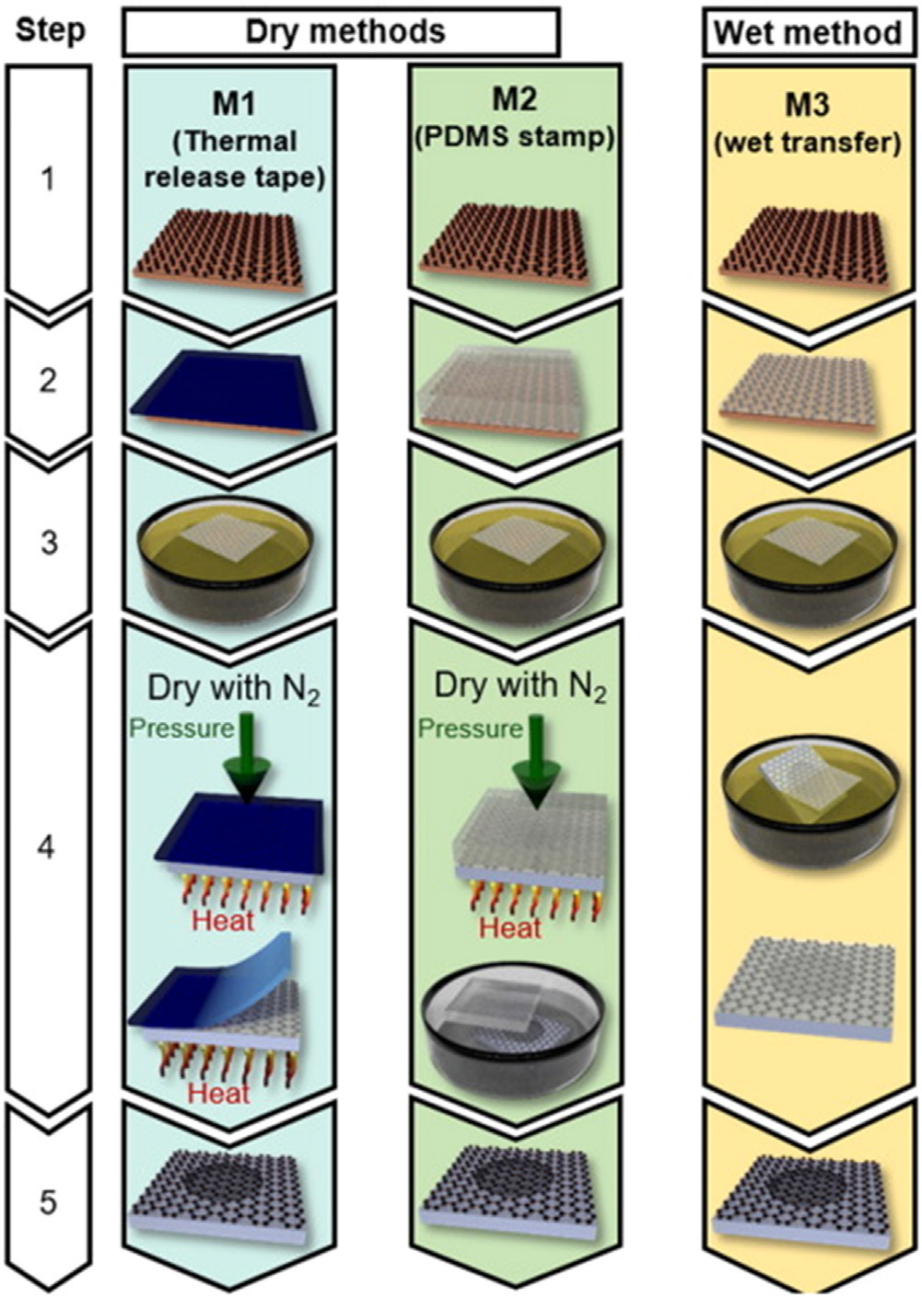
**Overview of the graphene transfer process (steps 1–5): (a) method M1 (dry transfer), (b) method M2 (dry transfer), and (c) method M3 (wet transfer) [**191**]**.

Apart from photonic applications, graphene is a highly suitable material for membrane-based nanoelectromechanical systems (NEMS). Wagner et al. explored three approaches for directly transferring CVD graphene onto pre-fabricated micro-cavity substrates, assessing each method's effectiveness in producing large-scale, high-quality, free-standing graphene membranes [191]. Three methods for transferring CVD-grown graphene from copper foil to substrates were explored: two dry transfer methods, thermal release tape (M1) and PDMS stamp (M2), and one wet transfer method (M3). In dry transfer, no liquids are involved during the process, unlike wet transfer, where liquid plays a role in graphene detachment. Fig. 9 outlines the steps for each method, all of which have been applied successfully to various substrates, though challenges arise when working with substrates containing holes for suspended graphene membranes.

In M1, PMMA is spin-coated onto graphene on copper foil (Fig. 9a, step 1), followed by the application of thermal release tape to provide support (Fig. 9a, step 2). The copper was etched, cleaned, and rinsed (Fig. 9a, step 3), after which the stack was dried with nitrogen. It was then pressed onto the substrate with graphene facing down, heated to enhance adhesion, and the thermal tape was peeled off, leaving graphene on the substrate (Fig. 9a, step 4). A photoresist layer was applied for channel patterning, followed by oxygen plasma etching, and the PMMA and graphene were removed in acetone (Fig. 9a, step 5). In M2, graphene on copper was covered with PMMA, and a thick photoresist was added, followed by pressing a PDMS stamp onto the stack (Fig. 9b, steps 1 and 2). After copper etching and cleaning, the stack was lifted and dried. The graphene was then pressed onto the target substrate (Fig. 9(b), step 3), and the photoresist layer was dissolved in isopropyl alcohol (Fig. 9b, step 4). Oxygen plasma etched the graphene and PMMA, and the stack was cleaned in acetone (Fig. 9b, step 5). M3 begins by spin-coating PMMA onto graphene on copper (Fig. 9c, steps 1 and 2). After etching and cleaning, the graphene was floated in deionized water (Fig. 9c, step 3). The target substrate was used to lift the graphene, which was then dried and heated to improve adhesion (Fig. 9c, step 4). Patterning was done, and after oxygen plasma etching, the polymers were removed in acetone (Fig. 9c, step 5). These methods offer effective approaches for transferring graphene to substrates with micro-cavities, crucial for fabricating stable suspended graphene membranes for advanced applications [191].

### 4.3 Printing techniques

Printing methods, including inkjet and screen printing, offer scalable and cost-effective alternatives for graphene integration, particularly in the development of flexible and disposable sensors [192]. These techniques utilize graphene-based inks, which contain graphene flakes dispersed in a solvent, allowing direct application onto sensor substrates. Inkjet printing, known for its precision, enables patterned deposition of graphene, making it suitable for creating defined sensor regions and microscale designs [193]. Screen printing, on the other hand, can deposit thicker graphene layers that enhance light absorption for photonic sensing applications [194]. However, printed graphene films may exhibit lower conductivity and uniformity than CVD-grown films, posing challenges for high-performance applications. Researchers are actively refining printing techniques to improve film quality, achieving conductivity and uniformity levels that approach those of other integration methods [195].

Each of these integration methods offers distinct advantages, and often multiple techniques are combined to optimize graphene’s functionality within specific sensor designs. These techniques allow graphene to be incorporated into various photonic architectures, from optical waveguides to fiber-optic sensors, expanding the range and adaptability of graphene-based photonic sensors. By leveraging these integration methods, researchers can harness graphene’s exceptional properties to create highly sensitive, reliable, and versatile sensors for diverse applications in medical diagnostics, environmental monitoring, and industrial process control.

Inkjet printing offers an efficient and scalable technique for organizing colloidal materials into targeted patterns without requiring vacuum conditions or lithographic steps. Two-dimensional (2D) nanosheets are especially suitable for printed electronics, as they support stable ink formulations compatible with solution processing and span diverse electronic properties, including metallic, semiconducting, and insulating types. Their surfaces, free from dangling bonds, allow for atomically thin films with active electronic properties and van der Waals interfaces, which reduce junction resistance. Song et al. presented thin-film transistors created entirely through inkjet printing, using electrochemically exfoliated graphene, MoS₂, and HfO₂ as the conductive electrodes, semiconducting channel, and high-k dielectric layer, respectively [196]. Notably, the HfO₂ dielectric layer was developed via a two-step process involving the electrochemical exfoliation of HfS₂ (a semiconductor) and subsequent thermal oxidation, addressing the challenge of exfoliating insulating materials. As a result, the fully inkjet-printed 2D nanosheets with varied electronic functionalities enable high-performance thin-film transistors, achieving notable field-effect mobilities.

**Fig. 10:**
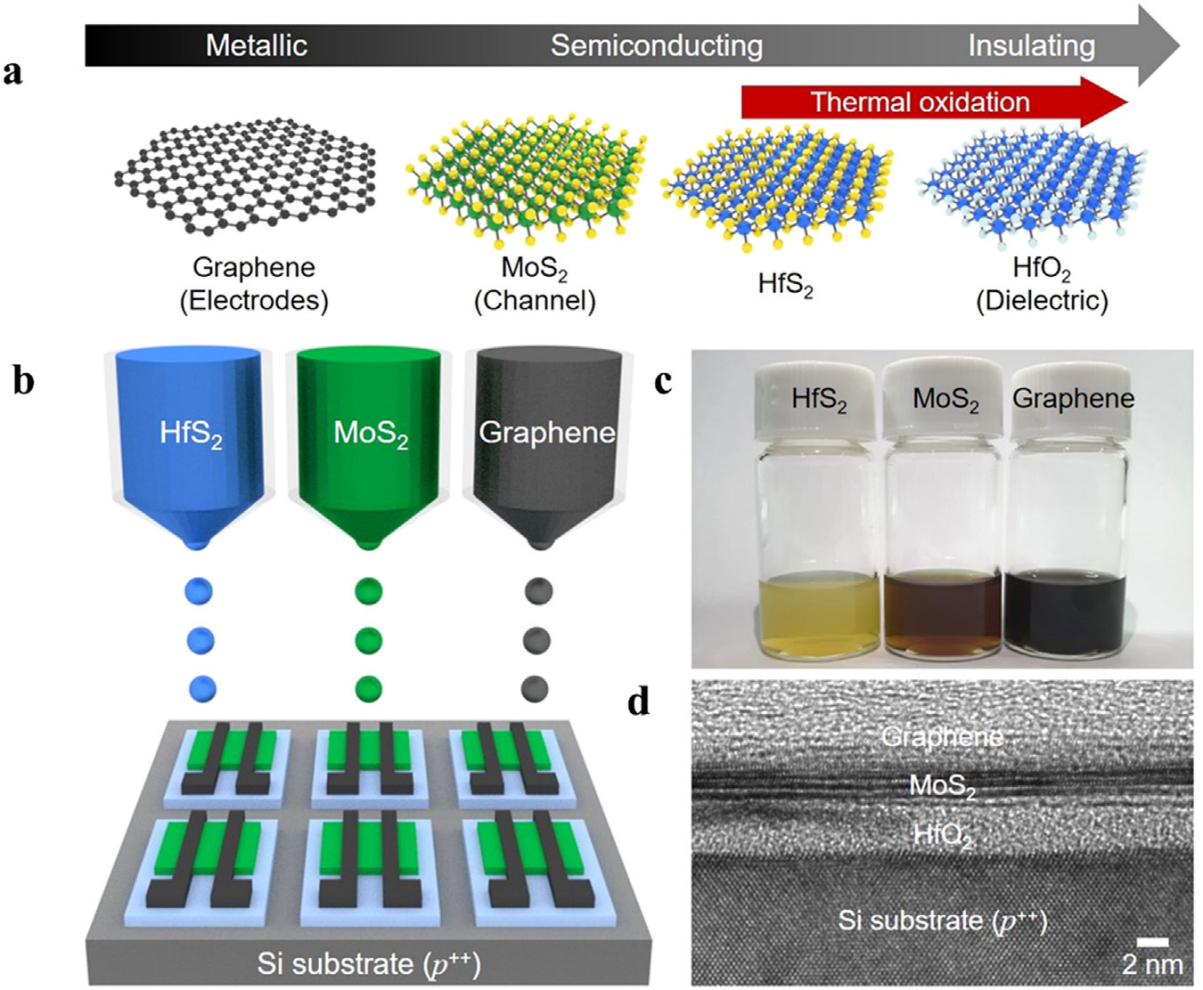
**(a) Detailed atomic structures and electronic properties of the ink materials, highlighting graphene with metallic conductivity, MoS₂ as a semiconducting layer, and HfO₂ as an insulating material derived from the oxidation of HfS₂, (b) Illustration of the inkjet printing process, (c) Optical image of the inks, showing MoS₂ and HfS₂ dispersed in isopropanol (0.5 mg/mL) and graphene in a mixture of ethanol and DMF (10:1 ratio, 0.5 mg/mL), (d) Cross-sectional TEM image showcasing the layered architecture and intricate material interfaces within the fully fabricated inkjet-printed transistor [**196**]**.

The atomic structure of 2D nanosheets assembled into thin-film electronic devices exclusively through inkjet printing is shown in Fig. 10a. Using a molecular intercalation-assisted electrochemical exfoliation technique on van der Waals crystals, 2D nanosheets spanning a full spectrum of electronic properties were produced: graphene as a conductor, MoS₂ as a semiconductor, and HfO₂ (formed by thermal oxidation of HfS₂) as an insulator. These nanosheets were suspended in alcohol-based solvents chosen for optimal substrate wettability and rapid drying, enhancing printing efficiency. The nanosheets were sequentially printed onto a heavily doped (*p*++) Si wafer, enabling the fabrication of devices such as field-effect transistors (FETs) and photodetectors (Fig. 10b). Optical images of the inks reveal stable nanosheet dispersion without aggregation, while critical rheological properties such as surface tension and viscosity ensure reliable inkjet printing by preventing nozzle clogging and minimizing satellite droplet formation (Fig. 10c).

## 5 Performance metrics and comparison with conventional photonic sensors

This section explores the key advantages of graphene-coated photonic sensors in terms of sensitivity, response time, limit of detection, selectivity, and stability.

### 5.1 Sensitivity

The sensitivity of photonic sensors refers to their ability to detect small changes in the surrounding environment, such as variations in refractive index, molecular binding, or chemical composition [1]. Sensitivity is typically quantified as the shift in an optical signal (e.g., wavelength or intensity) per unit change in the measured parameter. Several mechanisms contribute to determining this sensitivity. Resonance-based mechanisms, such as LSPR or guided-mode resonance, rely on changes in the local dielectric environment to shift the resonance condition. Evanescent field interactions, commonly employed in waveguide or SPR sensors, enhance surface-level detection by confining light near the sensor surface [16,197]. Graphene-coated sensors offer substantially enhanced sensitivity compared to conventional photonic sensors. Thanks to graphene’s unique properties such as its high surface-to-volume ratio, excellent conductivity, and tunable electronic characteristics, these sensors exhibit heightened responsiveness to minute environmental changes. When integrated with optical waveguides, graphene interacts strongly with analytes, boosting light absorption and altering the evanescent field at the sensor interface. This allows graphene-coated sensors to detect low analyte concentrations with greater precision, a significant advantage over traditional sensors. For example, in moisture detection for transformer oil, graphene’s strong affinity for water molecules leads to detectable shifts in optical properties even at trace moisture levels [149]. In contrast, uncoated or conventional sensors may require higher analyte concentrations to achieve similar detection accuracy, limiting their usefulness in applications where high precision is essential.

### 5.2 Response time

The response time of graphene-coated sensors can vary, with improvements in some cases and drawbacks in others, depending on the specific application and environment. Graphene’s high conductivity and rapid charge-transfer capabilities can enable quicker response times by accelerating interactions between the sensor surface and analytes. In applications where graphene has a strong affinity for the analyte such as gas or biomolecule detection—the response time is often significantly reduced due to swift adsorption onto the graphene surface. However, factors like graphene thickness and layer quality can also impact response time; for instance, multi-layer graphene coatings may slow response times due to higher diffusion resistance. Additionally, in complex environments with multiple analytes, graphene’s high sensitivity could lead to signal saturation, which may slow the sensor’s return to baseline once the analyte concentration decreases. Careful optimization of the coating process and material properties can help mitigate these potential challenges.

### 5.3 Limit of detection (LOD)

LOD in photonic sensors defines the smallest measurable change in a target parameter such as refractive index, analyte concentration, or molecular binding that can be reliably distinguished from background noise. A lower LOD indicates a more sensitive sensor capable of detecting trace amounts of substances or minute environmental changes [198]. LOD is influenced by several factors, including the quality of the optical resonance (e.g., high Q-factor), signal-to-noise ratio, stability of the optical setup, and the intrinsic sensitivity of the sensing mechanism. Enhancements such as surface functionalization, use of high-index contrast materials, and integration of nanostructures (e.g., plasmonic nanoparticles or 2D materials like graphene) can significantly reduce the LOD by increasing the interaction between light and analyte [199]. Ultimately, achieving a low LOD is critical for applications requiring high precision, such as biosensing, environmental monitoring, and medical diagnostics. Graphene notably lowers the LOD for various analytes, allowing the sensor to detect substances at much lower concentrations than traditional photonic sensors. The strong interaction between graphene and light produces more pronounced changes in optical properties, such as shifts in refractive index or light absorption, even at very low analyte concentrations [200]. In chemical sensing applications, for instance, graphene-coated sensors can capture trace amounts of target molecules, producing a measurable change in the optical signal. Additionally, graphene can be doped or functionalized to target specific analytes, further lowering the LOD by increasing selectivity and affinity. This enables graphene-coated sensors to detect substances at parts-per-million or even parts-per-billion levels, which is often unachievable with uncoated sensors [201]. Such a low LOD is crucial in applications requiring early detection of trace analytes, like environmental monitoring or biomedical diagnostics [202].

### 5.4 Selectivity

Graphene significantly enhances the selectivity of photonic sensors by reducing cross-sensitivity to non-target substances. Through functionalization, graphene’s surface can be modified with specific chemical groups or molecules to create targeted interactions with particular analytes [203]. In gas sensing, for instance, graphene can be engineered to preferentially bind with certain gases while ignoring others, enabling accurate measurements even in complex, mixed-gas environments. This high selectivity minimizes interference from unwanted species, leading to more reliable data in applications with complex sample matrices [204]. The versatility of graphene functionalization, combined with its strong optical and electronic properties, makes it ideal for applications like food safety testing, medical diagnostics, and environmental monitoring, where cross-sensitivity and sample complexity pose significant challenges [205,206].

### 5.5 Stability and durability

Graphene coatings enhance the stability and durability of photonic sensors, contributing to long-term, consistent performance [137]. Graphene’s remarkable mechanical strength and chemical inertness make it resilient against environmental stressors, including temperature changes, chemical exposure, and physical wear, thus extending the sensor’s lifespan. In harsh environments such as industrial or outdoor settings, graphene acts as a protective layer, shielding underlying sensor components from degradation due to humidity, UV exposure, or corrosive substances [74,207]. Furthermore, graphene’s flexibility allows it to endure mechanical stress without cracking or delaminating, preserving the sensor’s performance over time. This durability reduces maintenance and calibration requirements, ensuring reliable, long-term performance [208]. While environmental stability may vary based on the quality of the graphene layer and deposition method, graphene’s contribution to sensor robustness makes it highly advantageous for applications that demand sustained accuracy and stability in challenging conditions [207,209].

## 6 Challenges and future perspectives

Fabricating graphene-enhanced photonic sensors presents significant technical challenges, particularly concerning the uniformity, scalability, and reproducibility of graphene layers. These challenges are critical impediments to the development of robust, commercially viable devices capable of meeting the precision requirements in applications ranging from biomedical diagnostics to environmental sensing and optical communications [210]. The fabrication processes must ensure the consistency of graphene’s atomic structure, optical properties, and integration within photonic platforms. However, meeting these standards remains complex due to graphene’s delicate properties and the stringent requirements for device performance. To address these issues, advancements in fabrication techniques and quality control measures are being actively pursued, with promising solutions emerging across various domains of graphene production and photonic integration.

### 6.1 Uniformity challenges

Achieving atomic-level uniformity of graphene layers is essential for consistent optical and electronic performance in photonic sensors. Variations in layer thickness or atomic defects can drastically affect graphene’s optical absorption, conductivity, and photonic interactions, thereby reducing the sensor's precision and sensitivity. Non-uniform graphene layers lead to variability in key parameters such as refractive index, optical path length, and surface sensitivity, which can degrade the overall performance and reliability of photonic devices [211]. Current solutions for enhancing uniformity focus on optimizing CVD, a widely used technique that deposits graphene on metal substrates [184,212]. While CVD on copper can produce relatively high-quality monolayers, achieving perfect uniformity across large areas remains difficult due to challenges in controlling carbon precursor flow rates and maintaining substrate temperature [213]. To mitigate issues arising from the transfer process, new methods like direct graphene growth on dielectric substrates or epitaxial growth on silicon carbide (SiC) are being investigated, offering more stable and defect-free graphene films [214]. Advanced metrology techniques, such as in-situ Raman spectroscopy and atomic force microscopy (AFM), are also being integrated into the fabrication process to monitor and control layer quality in real-time, enabling adjustments to achieve greater uniformity.

Inkjet printing of graphene-based materials is another highly promising method due to its ease of use and ability to create flexible, graphical patterns. However, a major challenge in this process is overcoming the coffee ring effect, which leads to uneven patterns and material deposition. Sun et al. introduced an innovative and simple pre-deposition strategy to improve the uniformity of graphene inkjet printing [215]. By first applying a thin layer of ethanol, a series of processes such as homogenization, solvent exchange, stretching, and air drying occurred, resulting in a more uniform deposition of graphene nanosheets with a denser structure. The printed pattern produced using this method showed a much flatter surface compared to traditional approaches. This strategy was then applied to create an interdigital capacitive pressure sensor on a polyethylene terephthalate (PET) substrate. The resulting sensor demonstrated exceptional performance, with high sensitivity (a 33% change in capacitance under 10,000 Pa), low detection limits (able to detect as little as 0.1 g), and outstanding stability. Notably, it also exhibited minimal thermal hysteresis and improved performance relative to sensors made using direct inkjet printing, due to its compact structure and reduced air content. This method provided a promising solution for the practical use of low-cost, high-performance graphene-based electronic devices produced via inkjet printing [215].

### 6.2 Scalability challenges

The scalability of graphene-based photonic sensor production is a significant barrier to commercialization. Techniques such as mechanical exfoliation and laboratory-scale CVD are not suitable for high-throughput, large-area production due to their low yield and labour-intensive processes. Scaling up production without compromising graphene quality is crucial for reducing costs and enabling broader industrial adoption [216]. Efforts to improve scalability include advancing roll-to-roll CVD processes, which enable continuous graphene growth on metal foils that can later be transferred to photonic substrates [217]. Although this technique has shown potential for producing large-area graphene films, controlling quality over extended production runs remains a challenge, as variations in film thickness, crystallinity, and defect density can emerge. Direct deposition methods that grow graphene directly on photonic substrates (bypassing transfer processes) are under investigation as well, including plasma-enhanced CVD and other bottom-up approaches using carbon-based precursors [218]. Additionally, solution-based techniques, such as graphene oxide (GO) reduction and inkjet printing of graphene-based inks, offer alternative pathways to scalable production [219]. While solution-based approaches typically result in lower-quality films, recent advances in reducing defects and improving uniformity make them promising candidates for scalable photonic sensor applications where ultra-high precision is not a primary requirement [220].

2D electronic sensors, while easily integrated into existing water infrastructure, often face challenges related to inconsistent performance across devices. This variability—stemming from the absence of a reliable method to identify faulty units based solely on electronic characteristics—undermines accuracy and slows real-world deployment. To address this, Maity et al. presented a scalable approach combining wet transfer techniques, impedance and noise characterization, and machine learning to fabricate and assess graphene-based field-effect transistor (GFET) sensor arrays [221]. This integrated method enabled early detection and elimination of defective devices, improving overall reliability. These GFET sensors demonstrated real-time, simultaneous detection of heavy metal ions (lead and mercury) and *E. coli* bacteria in flowing tap water. This work established a robust quality control strategy that advances the practical application of electronic sensors for environmental monitoring [221].

### 6.3 Reproducibility challenges

Reproducibility in the fabrication of graphene-enhanced photonic sensors is critical, as even small variations in graphene’s structure or properties can lead to inconsistencies in sensor performance. Issues such as defect density, layer uniformity, and variations in optical and electronic characteristics arise from inconsistencies in the graphene synthesis and transfer processes. These inconsistencies lead to unpredictable shifts in sensor response, compromising the reliability needed for precise applications like biosensing, where target specificity and repeatability are essential [222].

To address reproducibility challenges, research has focused on establishing standardized fabrication protocols that tightly control environmental conditions, precursor purity, and processing parameters across synthesis and integration stages [223]. Automation in fabrication, including robotic-assisted transfer processes and automated deposition systems, is also reducing human-induced variability, thereby increasing the consistency of graphene layer properties [224]. Additionally, data-driven approaches such as machine learning are being applied to optimize fabrication parameters dynamically. Machine learning (ML) models analyze real-time data during fabrication, identifying process deviations that may affect reproducibility and enabling corrective adjustments to optimize device yield and reliability [225,226]. These automated and intelligent approaches are proving valuable in producing reproducible graphene photonic devices, particularly for applications where sensor precision and reliability are paramount [227].

**Fig. 11:**
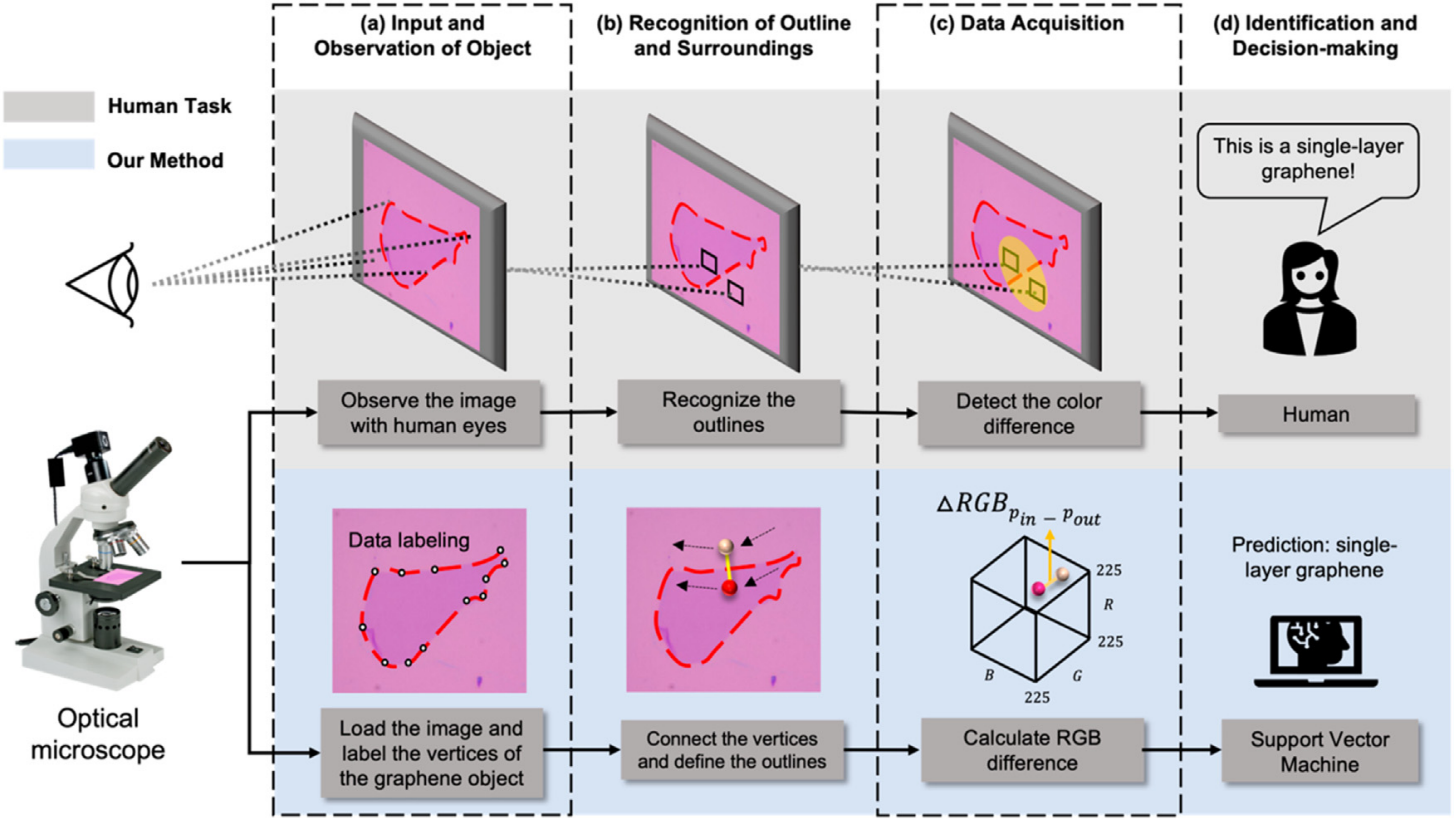
**Flowchart of our algorithm, designed to mimic how humans identify graphene layers: (a) Locate graphene objects: Identify graphene objects in an optical microscope image (requires initial labeling), (b) Detect outlines: Observe the outlines of these objects, (c) Compare color variations: Analyze color differences around the outlines, (d) Determine layers: Decide the number of graphene layers based on the extent of color differences [**225**]**.

Traditional techniques for identifying single-layer graphene, such as optical microscopy and Raman spectroscopy, are widely used but can be both exhaustive and costly. Yang et al. introduced an efficient and cost-effective alternative inspired by the perceptive ability of skilled researchers, who can often distinguish single-layer graphene by noting subtle color contrasts between graphene flakes and the substrate in optical microscope images [225]. This method emulated this human intuition through ML and targeted data analysis. To develop this approach, 300,000 pixel-level color difference data points were collected from 140 graphene flakes across 45 optical microscope images. The mean and standard deviation of these color differences were calculated for each flake, creating a robust dataset to train ML model. This model demonstrated strong performance, achieving F1-scores exceeding 0.90 and 0.92 for identifying 60 and 50 flakes on green and pink substrates, respectively (Fig. 11) [225]. This ML-driven system provided a scalable solution for graphene layer detection in various experimental settings, substantially reducing the need for traditional, laborious methods. This streamlined approach conserved both time and resources and opens new possibilities for classifying the properties of other two-dimensional materials, contributing to advancements in nanotechnology research.

Future research in graphene-coated photonic sensors should focus on several promising areas to enhance their performance and expand their applicability. One such area is the development of multi-layer graphene structures, which could offer superior electrical conductivity and optical response compared to single-layer graphene [228]. Multi-layer graphene has the potential to enhance sensor sensitivity by improving the interaction with light and providing greater mechanical stability [229]. Additionally, the exploration of hybrid materials, such as graphene-oxide composites, presents opportunities for combining the unique properties of graphene with the stability and functional versatility of other materials [230,231]. These hybrid systems could offer enhanced chemical stability, tunable optical characteristics, and improved interaction with external stimuli, thereby expanding the range of detectable analytes. Another avenue for future research is the design of novel sensor architectures that fully exploit the two-dimensional nature of graphene, as well as its ability to support SPRs and other optoelectronic phenomena. This could lead to the development of sensors with unprecedented sensitivity and selectivity, particularly for applications in environmental monitoring, healthcare diagnostics, and biochemical sensing. Furthermore, advancements in flexible and wearable sensor technologies could broaden the scope of graphene-coated photonic sensors for use in personalized medicine and portable sensing devices.

One-dimensional (1D) and two-dimensional (2D) materials are commonly used to create conductive networks on flexible substrates, forming the foundation of strain sensors in wearable electronics. However, limitations such as small contact areas and misalignment between layers have hindered the progress of flexible strain sensors utilizing 1D or 2D materials. To overcome these challenges, Li et al. introduced a hybrid strategy that combines 1D carbon nanotubes (CNTs) with 2D graphene nanoplatelets (GNPs) [232]. The resulting strain sensor, built from a CNT-GNP hierarchical structure, demonstrated excellent sensitivity and tunability. It can be stretched beyond 50% of its original length, with a high gauge factor of 197 at 10% strain, and showed significant recoverability after being stretched by 50%. This performance was attributed to the enhanced resistive behavior when subjected to strain. Furthermore, the GNP-CNT hybrid thin film delivered highly consistent responses over >1000 cycles of loading, ensuring long-term durability. This durability arose from the reinforcement of the conductive GNP networks through CNT hybridization [232].

Moreover, ultrasensitive resistive strain sensors (RSS) have gained significant attention due to their multifunctional potential. However, most of these sensors rely on cracked conductive metals, which experience performance degradation from structural deformation over repeated cycles. While new sensor designs have been proposed to address this issue, achieving both high stability and ultra-sensitivity remains a major challenge. Na et al. introduced a novel vertical graphene (VG)-based RSS that not only offered exceptional sensitivity (gauge factor greater than 5000) but also demonstrated remarkable durability (over 10,000 cycles) and resilience, making it ideal for various applications [233]. By incorporating carefully engineered cracks in a tufted network structure, the sensor achieved highly reversible resistance changes, with the ability to recover even after the current path is broken. This is confirmed through in situ microscopic monitoring. Furthermore, when integrated with a wireless sensing system, the VG-based RSS excelled in timbre recognition tasks. These findings provided valuable insights into the design of future mechanosensing systems and positioned VG as a promising material for next-generation flexible sensor technologies [233].

The scalability of graphene-coated photonic sensors is intricately linked to advancements in large-scale graphene production [217]. As previously mentioned, the high cost of graphene remains a significant barrier to its commercial viability. For graphene-based photonic sensors to become widely adopted, cost-effective and scalable methods for producing high-quality graphene must be developed. This includes optimizing synthesis techniques such as roll-to-roll processing for continuous graphene production or enhancing the efficiency of CVD methods. In addition to scaling up production, the integration of graphene sensors into existing photonic systems must be streamlined to ensure that they can be mass-produced without incurring prohibitive costs. The commercialization of these sensors will depend not only on overcoming production challenges but also on demonstrating their superior performance relative to conventional sensing technologies. If graphene-coated photonic sensors can deliver substantial improvements in sensitivity, response time, and miniaturization, they could find applications across a wide array of industries, including healthcare, environmental monitoring, telecommunications, and security. However, extensive validation and standardization efforts will be required to ensure their reliability, reproducibility, and robustness in real-world applications, and to meet the regulatory requirements for mass-market deployment.

## 7 Conclusion

In conclusion, graphene-coated photonic sensors represent a transformative advancement in the field of optical sensing, combining the exceptional optical, electrical, and mechanical properties of graphene with the precision and versatility of photonic technologies. The implication of these sensors lies in their ultra-high sensitivity, flexibility, and potential for miniaturization, making them valuable across diverse applications, from biomedical diagnostics and environmental monitoring to industrial process control and defence systems. Despite these advantages, limitations such as complex fabrication processes, graphene stability, and scalability challenges remain. Addressing these limitations requires innovative approaches in material engineering, device integration, and surface functionalization, along with a commitment to cost-effective manufacturing techniques. Future research should focus on overcoming these obstacles, particularly through enhanced graphene synthesis and hybrid material design, to unlock the full potential of graphene-coated photonic sensors.

As research progresses, these sensors are likely to play a progressively vital role in precision sensing, paving the way for a new generation of high-performance, versatile photonic devices across a range of high-impact fields. The future of graphene-coated photonic sensors holds transformative potential in fields like healthcare, environmental monitoring, and communications. Graphene’s unique properties—high conductivity, flexibility, and mechanical strength—enhance sensor performance by enabling sensitive, fast, and adaptable hybrid systems when combined with other materials. In healthcare, these sensors could enable real-time detection of biomolecules and diseases, while in environmental monitoring, they could detect pollutants and greenhouse gases with high precision. Additionally, graphene's capability to handle terahertz frequencies may drive advancements in ultrafast data transmission and photonic computing. As research progresses, graphene’s scalability and low-cost production make it key to next-generation sensors that could revolutionize various industries.

## CRediT authorship contribution statement

**Muhammad Ali Butt:** Project administration, Resources, Software, Supervision, Validation, Visualization, Writing – original draft, Writing – review & editing, Conceptualization, Data curation, Formal analysis, Funding acquisition, Investigation, Methodology.

## Declaration of competing interest

The authors declare that they have no known competing financial interests or personal relationships that could have appeared to influence the work reported in this paper.
